# COVID-19 Vaccination Status, Attitudes, and Values among US Adults in September 2021

**DOI:** 10.3390/jcm11133734

**Published:** 2022-06-28

**Authors:** Matthew Z. Dudley, Benjamin Schwartz, Janesse Brewer, Lilly Kan, Roger Bernier, Jennifer E. Gerber, Haley Budigan Ni, Tina M. Proveaux, Rajiv N. Rimal, Daniel A. Salmon

**Affiliations:** 1 Institute for Vaccine Safety, Johns Hopkins Bloomberg School of Public Health, 615 N. Wolfe Street, Baltimore, MD 21205, USA; janesse@23-four.com (J.B.); tproveau@jhu.edu (T.M.P.); dsalmon1@jhu.edu (D.A.S.); 2 Department of International Health, Johns Hopkins Bloomberg School of Public Health, 615 N. Wolfe Street, Baltimore, MD 21205, USA; haley.ni@cdph.ca.gov; 3 Fairfax County Health Department, 10777 Main St., Fairfax, VA 22030, USA; benjamin.schwartz@fairfaxcounty.gov; 4 National Association of County and City Health Officials, 1201 Eye Street, NW, 4th Floor, Washington, DC 20005, USA; lkan@naccho.org; 5 Independent Researcher, Aiken, SC 29803, USA; rogbernier@gmail.com; 6 RTI International, 701 13th Street NW, Suite 750, Washington, DC 20005, USA; jen.gerber07@gmail.com; 7 Department of Health, Behavior and Society, Johns Hopkins Bloomberg School of Public Health, 615 N. Wolfe Street, Baltimore, MD 21205, USA; rimal@jhu.edu

**Keywords:** COVID-19, vaccine, vaccination, vaccine hesitancy

## Abstract

Background: The Delta COVID-19 variant caused a resurgence in cases and deaths during the summer of 2021, particularly among the unvaccinated, highlighting the need to increase vaccine coverage. We describe a survey conducted in September 2021, in the midst of the Delta variant surge, after the FDA fully approved Pfizer-BioNTech’s vaccine for ages 16+ and issued an emergency use authorization for ages 12–15. Methods and Findings: US adults were surveyed to measure COVID-19 vaccination status, intentions, attitudes, values, and trust in public health authorities. More than three-quarters (77%) reported receiving at least one dose of COVID-19 vaccination. Of the unvaccinated, 6% intended to vaccinate, 40% were unlikely to ever vaccinate, and 55% remained uncertain. Most of the unvaccinated were <45 years old (62%), without a bachelor’s degree (83%), earning less than $85,000 annually (74%), and Republican/Independent (66%). Concerns among the unvaccinated-yet-still-uncertain included the vaccines’ safety (86%), speed of development (86%), and suspicion of government (79%) and pharmaceutical companies (69%). Most (86%) of the unvaccinated reported they would not vaccinate if mandated by their employer. About one third (34%) of the unvaccinated reported facing at least one barrier to vaccination. Conclusion: More than half of unvaccinated adults remained uncertain about COVID-19 vaccination, indicating an opportunity to support their decision making. Public health must increase easy and equitable access to vaccination and renew efforts to provide unvaccinated populations access to information from trusted sources.

## 1. Introduction

As of December 2021, three vaccines against Coronavirus Disease 2019 (COVID-19) have been authorized or approved by the United States (US) Food and Drug Administration (FDA). Pfizer-BioNTech and Moderna both received an Emergency Use Authorization (EUA) for adults in December 2020 [1,2], and Johnson & Johnson (J&J) received an EUA for adults in February 2021 [3]. Pfizer-BioNTech also received an EUA for adolescents in May 2021 [4], children 5–11 years old in October 2021 [5], and full approval for adults in August 2021 [6]. After peaking during the winter of 2021, rates of COVID-19 cases and deaths dropped precipitously in the spring as vaccine supply caught up to demand and a large portion of the population received newly available vaccines [7]. However, cases and deaths surged again as summer turned to fall, due to the proliferation of the more contagious Delta variant, primarily among the unvaccinated [8]. Vaccines remained quite effective against Delta, especially for severe disease and death, although slightly less effective than against the original strain [9,10,11]. At least 94% of reported US COVID-19 hospitalizations and deaths in 2021 occurred in unvaccinated persons [12]. Improving vaccine acceptance remains a key component of controlling the pandemic and returning to normal social and economic activities. 

A key feature of the COVID-19 pandemic has been change: change in the rates of infection with multiple surges occurring in communities; change in the circulating variants; change in the availability of countermeasures; changes in medical and public health knowledge; and reflecting all this, changes in what is communicated to the public through various media. These rapid changes have likely led many people to adapt their views, behaviors, and information consumption habits, accordingly, necessitating regular updates to public survey data assessing these topics. 

We conducted an initial national panel survey in December 2020 (prior to the first EUA) to measure COVID-19 vaccine attitudes, values, and intentions among US adults [13]. We found half of the adult US population likely intended to receive the vaccine once available, while 40% were uncertain and 10% unlikely to ever vaccinate. Intent to vaccinate was lower among Black Americans, women, young adults, Republicans, those living in a non-metro area, those with lower income, those with larger households, and those without a bachelor’s degree. Trust in local, state, and federal public health authorities and confidence in vaccines were highly associated with intending to receive COVID-19 vaccines.

Herein, we describe findings from a second national panel survey, conducted in September 2021. We captured another snapshot of COVID-19 vaccine attitudes, values, and intentions among US adults, but at a very different stage in our fight against this pandemic as compared to the first survey, with the survey topics updated accordingly. Our main focus is on those still unvaccinated—who they are, their attitudes and values, their trust in institutions and public health officials, and their reasons for not vaccinating yet. We elucidated factors associated with vaccination and intentions to vaccinate in the future. We also explored differences by race and ethnicity, and looked for changes in trust in public health authorities over time by political affiliation. 

## 2. Materials and Methods

### 2.1. Panel Survey

This second national panel survey was conducted online in English and Spanish among US adults (18+ years of age) between 24 August and 8 September 2021. As with the first survey [13], a representative sample was selected through the Ipsos KnowledgePanel [14], a probability-based web panel with about 60,000 members initially recruited by mail. Of the 4690 panel members selected and emailed an invitation to complete this survey, 2546 did so (54% completion rate). To increase the sample’s representativeness to the US population, households without internet access were given tablet computers and internet access; of the 164 selected panel members who did not have access to the Internet, 75 completed the survey. Hispanic Americans were supplementally recruited through random digit dialing of area codes with concentrated Hispanic populations. Enrollment quotas ensured the sample’s sociodemographic distribution approximated that of the US, with 50% oversampling of Black and Hispanic respondents.

This work was considered to fall under public health surveillance and not human subject research by the Johns Hopkins Bloomberg School of Public Health Institutional Review Board.

### 2.2. Survey Content

The survey was largely based on the Health Belief Model [15] and the Social Ecological Model [16]. The survey measured self-reported COVID-19 vaccination status, as well as hesitation experienced among the vaccinated and intentions among the unvaccinated. Vaccinated respondents selected one of the following answer choices: I got it as quickly as I could without any hesitation, I had some hesitation but got it anyway, I had a lot of hesitation but got it anyway. Unvaccinated respondents selected one of the following answer choices: definitely get it as soon as possible, probably get it as soon as possible, probably get it but not as soon as possible, probably not get it, definitely not get it. 

The survey measured three constructs: (1) confidence in vaccines (6-question scale); (2) trust in the Centers for Disease Control and Prevention (CDC) (14-question scale); and trust in local and state health departments (HDs) (14-question scale). The trust scales were validated by study authors in previous work [17]. The survey measured attitudes about COVID-19 disease and vaccines, such as perceived disease susceptibility, disease severity (among unvaccinated), mask wearing, importance of vaccines to control the pandemic, and requirements for sharing personal information to get the vaccine (among unvaccinated). Self-reported influenza vaccination coverage and cumulative COVID-19 disease prevalence (ever having COVID-19 disease) were also captured. Sociodemographic characteristics including gender, race, ethnicity, age, education, region, metropolitan statistical area (MSA), income, employment status and political affiliation were available for all panel members. Several survey items we hypothesized may change over time (cumulative disease prevalence, disease susceptibility, importance of vaccines to control the pandemic, influenza vaccine coverage, all three construct scales) were measured among the full sample in both the initial December 2020 and this September 2021 survey to allow for comparison. Important new topics added to this survey that were not in the initial survey included: COVID-19 vaccine effectiveness and safety, COVID-19 disease and vaccines for children, trust in science, likelihood to receive booster doses (among vaccinated), and the potential impact on vaccine intent (among unvaccinated) of: employer vaccine requirements, financial incentives, coronavirus variants, known vaccine side effects, specific concerns, and barriers to receiving vaccines. 

### 2.3. Data Analyses

A ranking procedure was used to adjust the design weights so that the sample was weighted to the US population of adults at least 18 years old. Black and Hispanic respondents were oversampled and were down-weighted to reflect their proportion in the population. Further details on weighting were published previously [11].

For each of the three construct scales (confidence in vaccines, trust in CDC, trust in HDs), a composite, linear score was generated (Table S15). The numerator equaled the sum of responses to all answered items within the scale, where strongly disagree = 0, disagree = 1, agree = 2, strongly agree = 3. The denominator equaled the total possible score, accounting for missing variables, thus creating a scale from 0 to 100 (0 being complete disagreement and 100 being complete agreement) to facilitate comparisons across scales. Cronbach alpha coefficients for these scales were estimated to be between 0.87 and 0.93, indicating strong reliability (Cronbach alpha values range from 0 to 1, and values greater than 0.80 are generally considered good). The score was then dichotomized at the median creating “high” and “low” groups for each construct.

Unweighted and weighted univariate analyses were conducted for sociodemographic characteristics and vaccination/disease status. For all other variables, only weighted analyses were performed. Sociodemographic variables were cross-tabulated against COVID-19 vaccination status, hesitation (among vaccinated), and intention (among unvaccinated), and stratified by selected sociodemographic characteristics. Intention to vaccinate was reclassified as: (1) definitely or probably get it as soon as possible (Likely); (2) probably get it but not as soon as possible and probably not get it (Uncertain); and (3) definitely not get it (Unlikely). To facilitate straightforward analyses and interpretation, Likert scale response options (strongly agree, agree, disagree, strongly disagree, don’t know) were dichotomized to agree vs. disagree (don’t know, when included as an option, was coded as disagree), and other scale response options (e.g., very important, important, not very important, not at all important) were similarly dichotomized to reflect affirmative vs. negative (e.g., important vs. not important). Weighted proportions for variables assessed among the entire sample at both survey timepoints (December 2020 and September 2021) were compared to assess changes over time. 

For all weighted proportions, Taylor-linearized variance estimation was used to estimate standard errors. For all cross-tabulations and comparisons between timepoints, *p*-values were estimated using Pearson chi-squared proportion test at significance level of α = 0.05. Bivariate odds ratios were estimated using glm family (logit) with binary dependent variables for vaccination status, hesitancy to vaccinate (among the vaccinated), and intentions to vaccinate (among the unvaccinated), and binary independent variables for affirmative responses to select survey items (or dummy indicator variables for categorical sociodemographic characteristics). Data were analyzed using Stata, version 16 [18].

## 3. Results

### 3.1. Study Population and Survey Weighting

Of the 4690 panel members fielded the survey, 2546 completed it (54% completion rate). Sociodemographic characteristics and vaccination status of the survey respondents (N = 2546), unweighted and weighted, are presented in Table 1. Weighting had a limited impact other than by race and ethnicity (with oversampling of Black and Hispanic populations), given the panel was designed to represent the US population. Weighted data are generalizable to the US adult population.

### 3.2. Vaccination Status

Over three-quarters (77%) of the weighted sample reported having received at least one dose of COVID-19 vaccine (Table 2). Of these, 89% received two doses of the mRNA vaccine (Pfizer-BioNTech or Moderna), 4% received one dose of the mRNA vaccine, and 7% received the one-dose Johnson & Johnson vaccine. Non-Hispanic Black Americans had lower odds of being vaccinated than non-Hispanic White Americans (odds ratio (OR): 0.72; 95% Confidence Interval (CI): 0.54–0.96); no difference was seen between Hispanic Americans and non-Hispanic White Americans, and non-Hispanic Americans of other race/ethnicity (of whom 72% were Asian Americans) had higher odds of being vaccinated than non-Hispanic White Americans (OR: 2.09; 95%CI: 1.18–3.70). Other sociodemographic factors negatively associated with vaccination included: younger age, less education, living in the southern US (vs. northeastern US), living in a non-metro area, lower income, larger household/more children, and Republicans (vs. Democrats). 

Having gotten the flu vaccine within the past year was positively associated with COVID-19 vaccination (OR: 9.00; 95%CI: 6.72–12.06) and having ever had COVID-19 disease was negatively associated with vaccination (OR: 0.40; 95%CI: 0.30–0.53). Positive vaccine and prevention (e.g., mask wearing) attitudes were strongly positively associated with vaccination, and negative vaccine attitudes were strongly negatively associated with vaccination. For example, those who believed COVID-19 vaccines important in stopping the spread of infection had nearly 40 times higher odds of vaccinating compared to those who did not (OR: 39.10; 95%CI: 27.47–55.67), and those worried about COVID-19 vaccine safety had about 25 times lower odds of vaccinating compared to those who were not (OR: 0.04; 95%CI: 0.03–0.06). Vaccinating was also positively associated with discussing vaccination with a healthcare provider (OR: 1.60; 95%CI: 1.24–2.06), provider encouraging vaccination (OR: 7.85; 95%CI: 4.89–12.59), and trust in CDC (OR: 11.40; 95%CI: 8.17–15.91), HDs (OR: 5.75; 95%CI: 4.30–7.69), and science (OR: 5.21; 95%CI: 3.70–7.34). Seeking health information from healthcare providers (ORs: 1.66–2.66), news outlets (OR: 2.07; 95%CI: 1.14–3.77), and the internet (OR: 1.30; 95%CI: 1.03–1.65) was positively associated with vaccination, while not seeking health information was negatively associated with vaccination (OR: 0.40; 95%CI: 0.31–0.52) (Table S1). 

Although nearly two thirds (65%) of those vaccinated had no hesitation prior to doing so, nearly one quarter (24%) had some hesitation, and about one tenth (11%) had a lot of hesitation (Table S2). Most vaccinated persons (87%) indicated they were likely to receive a booster dose if recommended, particularly among those who initially vaccinated without hesitation (97%) as compared to those who initially vaccinated with some hesitation (79%) or a lot of hesitation (46%) (*p* < 0.01). 

### 3.3. Characterizing the Unvaccinated

Most of the unvaccinated were under 45 years of age (62%), without a bachelor’s degree (83%), making under $85,000 annually (74%), and Republican or Independent (66%). Only 18% of the unvaccinated had received a flu vaccine within the past year. 

#### 3.3.1. Vaccine Intentions among the Unvaccinated

Although only 6% of the unvaccinated indicated they were likely to vaccinate as soon as possible and 40% were unlikely to ever vaccinate, over half (55%) were still uncertain (Table 3). Many of the attitudes and values associated with vaccination as described above were also associated with intention to vaccinate. In addition, those reporting believing it likely they will get COVID-19 disease over the next year had higher odds of being likely vs. unlikely to vaccinate (OR: 6.01; 95%CI: 2.24–16.18). The only associations between sociodemographic characteristics and vaccine intentions among unvaccinated were: Democrats had higher odds of being likely (OR: 4.31; 95%CI: 1.38–13.44) or uncertain (OR: 2.62; 95%CI: 1.39–4.91) vs. unlikely to vaccinate than Republicans, and Hispanic Americans had higher odds of being likely vs. unlikely to vaccinate than non-Hispanic White Americans (OR: 5.17; 95%CI: 1.78–15.06). 

#### 3.3.2. Barriers to Vaccination among the Unvaccinated 

Over half (59%) of those unvaccinated yet likely to vaccinate and about one third (34%) of those unvaccinated yet still uncertain reported facing at least one barrier to vaccination. Common barriers to vaccination reported were being unable to miss work (34% of likely, 19% of uncertain), vaccine availability conflicting with daily schedule (47% of likely, 12% of uncertain), not knowing how to register for an appointment (32% of likely, 11% of uncertain), and registering for an appointment being too difficult (23% of likely, 18% of uncertain). Relatively few reported not having transportation to vaccination locations (1% of likely, 7% of uncertain). 

#### 3.3.3. Potential for Mandates and Financial Incentives to Increase Vaccination

If their employer required COVID-19 vaccination, only 14% of the unvaccinated reported they would get vaccinated, including nearly half (49%) of those likely to vaccinate, 16% of those still uncertain, and 5% of those unlikely to vaccinate. Of the remaining 86% of unvaccinated who reported they would not vaccinate if required by their employer, 29% reported they would quit their job, 32% reported they would protest, and 43% reported they would consider legal action. If offered a $25–$100 gift card or a lottery ticket to be eligible to win at least $100,000, only 5 and 6% of the unvaccinated reported they would get vaccinated (25% and 19% of likely, 7% and 7% of uncertain, <1% and 2% of unlikely), respectively. 

#### 3.3.4. Reasons to Vaccinate among the Unvaccinated and Uncertain

Nearly one half of the unvaccinated yet still uncertain reported concerns about exposure to COVID-19 in public (46%) or belief in vaccines’ importance in stopping the spread of infection (50%). Between one fifth and one third of the unvaccinated and uncertain agreed that vaccines would protect them from strains currently circulating (34%) or potentially appearing in the future (29%), it was important for them to get vaccinated to avoid infecting their family (29%) or help get their community back to normal (20%), or that the government was acting in their best interest regarding COVID-19 (23%).

#### 3.3.5. Specific Concerns and Reasons Not to Vaccinate among the Unvaccinated and Uncertain

Most of the unvaccinated yet still uncertain worried about the safety of COVID-19 vaccines, whether for adults (68%), children (87%), or in general (86%). The most prevalent specific concerns about COVID-19 vaccines among the unvaccinated and uncertain included: not trusting the speed of their development (86%), their safety not being studied for a long enough period of time (81%), their newness (79%), recommendations being influenced more by politics than by science (79%), potential immediate side effects (74%) or allergic reactions (74%), safety concerns regarding ingredients (73%), drug companies making a lot of money (69%), and experimenting on people (61%). Other less prevalent but still relatively common concerns among the unvaccinated and uncertain included: having a health condition perceived to increase the risk of having a bad reaction to vaccination (32%), effects on fertility (26%), potential changes to DNA (24%), having to provide personal information to get it (23%), and some vaccines being made from aborted fetuses (17%). About half of the unvaccinated and uncertain reported those they trust did not want the vaccine (47%) or they had seen posts on social media that made them wary of the vaccine (54%). About four-fifths of the unvaccinated and uncertain wanted to wait and see what happens to others who get vaccinated (81%), or needed more time to learn and think about it (80%). 

#### 3.3.6. Differences among the Unvaccinated by Race and Ethnicity

Almost two thirds (66%) of unvaccinated non-Hispanic Black Americans and 71% of unvaccinated Hispanic Americans remain open to vaccination (either likely to vaccinate or uncertain), compared to 57% of non-Hispanic White Americans (Table 4). Unvaccinated Hispanic Americans (28–33%) and non-Hispanic Black Americans (28–38%) were more likely to report the aforementioned reasons to vaccinate than unvaccinated non-Hispanic White Americans (12–22%). Unvaccinated non-Hispanic Black Americans had over double the odds (OR: 2.08; 95%CI: 1.22–3.57) of reporting facing at least one barrier to vaccination compared to unvaccinated non-Hispanic White Americans; in particular, that registering for an appointment was too difficult (25% of unvaccinated non-Hispanic Black Americans vs. 10% of unvaccinated non-Hispanic White Americans). No differences were found by race or ethnicity on the impact of mandates or incentives on intention to vaccinate. 

Although most of the aforementioned specific concerns and reasons not to vaccinate did not differ by race or ethnicity, several notable differences were found. Non-Hispanic White Americans were more likely to perceive COVID-19 vaccines as new (85%), worry their safety was not studied for a long enough period of time (87%), and have safety concerns regarding ingredients (80%) compared to Hispanic Americans (74%, 80%, and 69%, respectively) and especially non-Hispanic Black Americans (66%, 69%, and 62%, respectively). Non-Hispanic White Americans were also more likely to perceive vaccine recommendations as influenced more by politics than by science (88%) and report those they trust did not want the vaccine (60%) than Hispanic Americans (75% and 38%, respectively) and non-Hispanic Black Americans (66% and 42%, respectively). Hispanic Americans were more likely to worry about potential immediate side effects (82%) than non-Hispanic White Americans (75%) and especially non-Hispanic Black Americans (59%). Hispanic Americans (27%) and non-Hispanic Black Americans (26%) were more likely to perceive not enough people of their race/ethnicity participating in vaccine studies than non-Hispanic White Americans (10%). 

Among the unvaccinated and uncertain, Hispanic Americans (56%) and non-Hispanic Black Americans (77%) were more likely to report reported concerns about exposure to COVID-19 in public than non-Hispanic White Americans (35%) (*p* < 0.01) (Table S14). Unvaccinated and uncertain Hispanic Americans (25%) and non-Hispanic Black Americans (37%) were also more likely to agree it was important for them to get vaccinated to help get their community back to normal than unvaccinated and uncertain non-Hispanic White Americans (16%) (*p* = 0.01).

### 3.4. Changes over Time

Between December 2020 and September 2021, confidence in vaccines (in general—not specific to COVID-19) increased 7% (*p* < 0.01) (Table 5). Cumulative self-reported COVID-19 disease prevalence increased 14% (from 5% to 18%), perceived likelihood of COVID-19 infection decreased 18% (from 37% to 20%), and perceived importance of COVID-19 vaccines in stopping disease spread decreased 6% (from 88% to 82%). However, trust in the CDC and HDs stayed roughly the same. The significance and direction of these changes mostly remained consistent when stratifying by political affiliation, although perceived importance of COVID-19 vaccines in stopping disease spread decreased 11% (from 85% to 74%) among Republicans while staying at 95% among Democrats. 

## 4. Discussion

This nationally representative panel survey provides an accurate estimation of COVID-19 vaccination status, intentions, attitudes, values, and trust in public health authorities among US adults. It was conducted in September 2021, in the midst of the Delta variant surge, after the FDA fully approved Pfizer’s vaccine for ages 16 and up [6], gave an EUA for ages 12–15 [4], and when an EUA for booster doses and primary dose vaccination for ages 5–11 years was on the horizon [5]. Although many of the findings of this survey are reflected elsewhere in the literature, this study provides a thorough assessment of topics not commonly addressed in other surveys, such as barriers to vaccination, attitudes towards vaccine mandates, and specific reasons to consider vaccinating among the unvaccinated.

More than three quarters (77%) of our nationally representative sample of US adults had received at least one dose of a COVID-19 vaccine. This aligns with data from Kaiser Family Foundation (KFF), which estimated 72% of adults had received at least one dose as of 22 September 2021 [19], and from CDC, which estimated 79% of adults had received at least one dose as of 20 October 2021 [7]. However, almost one quarter (23%) of US adults remained unvaccinated. Our data strongly associate vaccination with most sociodemographic characteristics assessed (including age, education, income, race/ethnicity, political affiliation, metro status, parental status, and household size), likely partly due to collinearity between sociodemographic factors, but also suggesting clustering of unvaccinated persons both geographically and socially. Geographic clustering of the unvaccinated has been recognized with both COVID-19 [20] and other vaccines [21,22]. Communities with low vaccine coverage fueled the Delta surge despite relatively high vaccine coverage nationally [8]. Repeated surges with COVID-19 variants demonstrate the continued need to improve vaccine coverage as long as the virus continues to spread, even when coverage is relatively high and cases are decreasing on a national or state level. 

Our December 2020 survey [13] was conducted prior to the first EUA for the Pfizer-BioNTech vaccine [1], but after widespread publicity suggested favorable safety profiles and high levels of efficacy (~95%) for both the Pfizer-BioNTech and Moderna vaccines. We found half of the adult US population reported intending to likely receive the vaccine once available, while 40% were uncertain and 10% were unlikely to ever vaccinate. Comparing these findings with current vaccine coverage estimates indicates that many who were unsure at first ultimately decided to get vaccinated despite their initial concerns. Immediately prior to vaccine availability, intent to vaccinate was relatively less frequent among Black Americans, women, young adults, Republicans, those living in a non-metro area, those with lower income, those with larger households, and those without a bachelor’s degree. Other than gender, these sociodemographic associations with intent remained associated with vaccine acceptance in this second survey. 

We hypothesized that certain vaccine attitudes and trust in CDC and HDs may change from December 2020 to September 2021, as vaccines were introduced, disease dynamics changed, and new government leadership took effect. We also anticipated some such changes may differ by political leanings, given the unfortunate politicization of the pandemic and the vaccine. Reassuringly, confidence in vaccines (in general) increased among both Republicans and (especially) Democrats. However, perceived importance of COVID-19 vaccines decreased substantially among Republicans (while remaining high among Democrats), suggesting continued polarizing effects of this politicization. Surprisingly, trust in CDC and HDs stayed roughly the same, albeit rising slightly for CDC among Democrats. Meanwhile, cumulative COVID-19 disease prevalence increased substantially for both Republicans and Democrats. 

As of September 2021, more than half of the unvaccinated remained uncertain, and over one third of the vaccinated reported overcoming hesitation prior to vaccinating, illustrating the potential to support decision making and increase vaccine coverage among those who have not yet ruled it out. Most of the remaining unvaccinated were younger, lower education, lower income, and Republican or Independent, indicating sociodemographic characteristics to target in outreach. However, there remained much heterogeneity among the unvaccinated, especially among the uncertain. Unvaccinated Democrats and Hispanic and Black Americans were more likely to be concerned about exposure to COVID-19 and remain open to and recognize the value of vaccination, than unvaccinated Republicans and White Americans, respectively. These differences by race/ethnicity and political affiliation are consistent with other studies [23,24,25], and indicate where limited resources may be most efficiently directed to increase vaccine coverage. Most of the unvaccinated worried about vaccine safety, including the uncertain. The most prevalent concerns about COVID-19 vaccines among the unvaccinated included their newness and side effects, as well as suspicion of government and pharmaceutical companies. These concerns are largely reflected elsewhere in the literature [19,26,27]. Very few of those unlikely to vaccinate but nearly half of the uncertain worried about disease exposure and believed vaccines effective or important. About one third of the uncertain believed vaccines would protect them from current and future strains, and recognized the importance of vaccinating to protect their family. Most of the unvaccinated wanted to keep waiting to see what happens to others getting the vaccine, especially the uncertain. Messaging and outreach should aim to support decision making among the uncertain by emphasizing the benefits of vaccination versus the risk of the disease and addressing concerns in an empathetic and comprehensible manner, leaving the door open for continued conversation and consideration. 

There is also still an opportunity to increase vaccine coverage through increased access. About one third of the unvaccinated reported facing at least one barrier to vaccination, including more than half of those intending to vaccinate soon (a group disproportionately Hispanic and Democrat). Most of these barriers were related to scheduling conflicts, namely being unable to miss work to vaccinate. Some of the unvaccinated also did not know how or found it difficult to register for an appointment. Black Americans had double the odds of reporting at least one barrier than White Americans, and in particular were more likely to find it difficult to register for an appointment. These reported barriers highlight the importance of continuing to expand easy and convenient access to vaccination, especially outside of normal working hours and vaccinating walk-ins without formal registration requirements. However, although reducing these barriers may alone be enough to vaccinate the relatively small population who are unvaccinated but intend to vaccinate soon, the much larger population who are uncertain will likely need both a reduction in barriers and additional decision-making support. 

COVID-19 vaccine mandates have already been widely implemented for employees in many healthcare facilities and universities, as well as government offices (federal, state, and local), large companies (via federal requirements), and some smaller companies (by their own choosing). A vaccine mandate has even been preemptively approved (once a BLA is granted) for school children in California [28]. These mandates may have already positively impacted vaccine acceptance [29]. While there is strong evidence supporting the net benefit of mandates for previous vaccines among school children [30] and healthcare facilities [31], the US does not have experience implementing vaccine mandates in situations where about a quarter of the population does not want the vaccine even after widespread free access. The highly politicized and polarized COVID-19 environment in the US may increase the risk of pushback to mandates. We found evidence of this potential for pushback, as only 14% of the unvaccinated said they would get vaccinated if required by their employer, many threatening to instead protest, consider legal action, or quit. We also found limited remaining potential of financial incentive programs, as only 5–6% of the unvaccinated reported they would get vaccinated if offered a gift card or lottery ticket. Our data may undersell incentive programs and (especially) mandates for several reasons. Firstly, at this point in the pandemic, many of those most likely to respond positively to incentives and mandates have likely already been vaccinated, perhaps influenced by such programs already implemented. Additionally, survey data may exaggerate the risk of pushback to mandates, as in the real world many may not follow through on anonymous threats made in a survey. However, experimental data also suggest COVID-19 vaccine mandates may have limited potential for positive impact and risk backlash [32]. 

The biggest limitation of this study is that it provides a snapshot at a single point in time. However, the survey was timed to capture a critical moment in the pandemic, measuring vaccination status during the height of Delta and after the first full FDA approval. We were also able to make some interesting comparisons to our earlier survey of participants recruited from the same panel, although we were limited in how many survey items we could measure at both survey timepoints, as we had to balance adding new items of interest while also keeping the survey within restrictions on length. Another limitation is the likelihood of strong correlation between many demographic factors examined (e.g., race and ethnicity, education, income). We chose to address this by performing stratified analyses instead of multivariate analyses, as often the interpretation of multivariate analyses can be difficult and without clear public health implications. Our data are also subject to the limitations of self-reporting. 

Despite many publicly available survey data assessing COVID-19 vaccine intentions and attitudes, many analyses have not undergone peer review [19,26,33,34,35]. Many of those that are published are of variable quality in terms of methodology and validity [23,25,27,36,37,38,39], only rarely using nationally representative samples to examine factors associated with vaccine intention with the ability to assess changes over time [40].

Strengths of our work include: the careful formulation of our survey and analyses, the quality of the Ipsos panel as a well-established nationally representative panel, the oversampling of Black and Hispanic populations to increase the precision of estimates when stratifying or comparing by race/ethnicity, the comparison of changes over time, and the thorough assessment of topics not commonly addressed in other surveys, such as barriers to vaccination, attitudes towards vaccine mandates, and reasons to consider vaccinating among the unvaccinated.

Public health immunization programs must achieve higher levels of COVID-19 vaccine uptake to effectively control the pandemic. This precise characterization of vaccine intentions, attitudes, and values of those still unvaccinated is meant to aid public health authorities at the local, state, and federal levels in their efforts to support decision making through their communication and outreach programs. Immunization programs should continue to expand convenient access to vaccination, especially in communities with low vaccine coverage. Messaging should continue to focus on these communities and explain how COVID-19 vaccines were created so quickly, emphasize the safety and importance of these vaccines, and remind of the remaining threat of the disease. Messages may be more trusted coming from sources outside of government and pharmaceutical companies. Mandates may create backlash and should be approached with caution. With renewed efforts to support decision making among the unvaccinated and uncertain, we still have the potential to control the pandemic. 

## Figures and Tables

**Table 1:** Sociodemographic Characteristics and Vaccination/Disease Status of the September 2021 Survey Sample: Unweighted and Weighted. The numbers of survey participants are listed by sociodemographic characteristics and vaccination/disease status. Survey weights were used so that the data were representative of US adults; specifically, Black and Hispanic respondents were weighted to adjust for oversampling performed to allow for stratified analyses with sufficient power. Unweighted and weighted percentages are presented for comparison.

	Unweighted	Weighted ^a^		Unweighted	Weighted ^a^
Sociodemographic Characteristics	N = 2546 (%)	%	Sociodemographic Characteristics	N = 2546 (%)	%
Gender			Household Annual Income		
Female	1292 (50.7)	51.5	<$50K	913 (35.9)	30.2
Male	1254 (49.3)	48.5	$50–85K	811 (31.9)	31.3
Age (years)			$85–150K	406 (15.9)	18.7
18–29	298 (11.7)	20.2	$150K+	416 (16.3)	19.8
30–44	617 (24.2)	25.4	Household Size		
45–59	704 (27.7)	24.2	1	471 (18.5)	16.6
≥60	927 (36.4)	30.2	2	916 (36.0)	35.3
Education			3	458 (18.0)	19.0
Less than high school	228 (9.0)	9.5	≥4	701 (27.4)	29.1
High school	619 (24.3)	27.8	Number of Children (Ages 2–17)		
Some college	842 (33.1)	27.7	0	1836 (73.5)	72.8
bachelor’s degree or higher	857 (33.7)	35.0	1	268 (10.7)	10.6
Race/Ethnicity			2	247 (9.9)	10.5
White, non-Hispanic	1034 (40.6)	63.2	≥3	148 (5.9)	6.1
Black, non-Hispanic	607 (23.8)	11.9	Political Affiliation		
Hispanic	789 (31.0)	16.5	Republican	524 (20.7)	25.8
Other, non-Hispanic	116 (4.6)	8.4	Democrat	1040 (41.1)	34.4
Of other: American Indian/Alaskan Native	7 (6.0)	9.3	Independent	691 (27.3)	29.6
Of other: Asian	60 (51.7)	72.2	Something else	276 (10.9)	10.2
Of other: Native Hawaiian/Pacific Islander	1 (0.9)	0.9	Physical Health		
Of other: 2+ races	48 (41.4)	17.6	Excellent	256 (10.1)	11.1
Region			Very good	905 (35.6)	36.5
Northeast	412 (16.2)	17.4	Good	941 (37.0)	36.5
Midwest	455 (17.9)	20.8	Fair	367 (14.4)	13.3
South	1054 (41.4)	38.0	Poor	75 (2.9)	2.7
West	625 (24.5)	23.9	Influenza Vaccination ^b^		
Metropolitan Statistical Area Status			Not Vaccinated	1137 (44.8)	45.2
Non-Metro	261 (10.3)	13.4	Vaccinated	1403 (55.2)	54.8
Metro	2285 (89.7)	86.6	COVID-19 Vaccination		
Employment Status			Not Vaccinated	523 (20.7)	23.0
Not Working	1013 (39.8)	37.9	Vaccinated	2002 (79.3)	77.0
Working	1533 (60.2)	62.1	Of vaccinated: J&J (1 dose)	138 (6.9)	7.5
COVID-19 Disease			Of vaccinated: mRNA (1 dose)	74 (3.7)	3.5
Never Had	1919 (82.9)	82.0	Of vaccinated: mRNA (2 doses)	1778 (88.9)	88.3
Ever had	396 (17.1)	18.0			

^a^ Weights produced using iterative proportional fitting so that respondents were weighted to represent US adults; Black and Hispanic respondents were weighted to adjust for the oversampling that was performed to allow for stratified analyses with sufficient power. ^b^ Respondents reported having received influenza vaccination within the past 12 months or not; these data were collected prior to the 2021–2022 influenza season, and so reflect the 2020–2021 influenza season.

**Table 2:** Frequency and Odds of COVID-19 Vaccination by Vaccine Attitudes, Trust in CDC and HDs, and Sociodemographic Characteristics. Numbers in the “Total” column indicate the percentage of the total weighted sample providing the September 2021 survey response in each row. Numbers in the “COVID-19 Vaccination” columns indicate the percentage of those whose COVID-19 vaccination status match that of the column header who provided the survey response in each row. The numbers in the “OR (95%CI)” column indicate the Odds Ratio of being vaccinated vs. unvaccinated by the survey response in each row, boldface indicating statistical significance *(p <* 0.05).

Survey Items	Total (%) ^a^	COVID-19 Vaccination, % ^b^		OR (95%CI) ^c^
		Unvaccinated	Vaccinated	
All (N = 2546)	100	23	77	
Constructs ^d^				
Confidence in vaccines	65	16	79	**19.97 (14.60–27.29)**
Trust in the Centers for Disease Control and Prevention (CDC)	47	11	58	**11.40 (8.17–15.91)**
Trust in local and state health departments (HDs)	46	17	55	**5.75 (4.30–7.69)**
Sociodemographic Characteristics				
Gender				
Female	52	53	51	ref ^k^
Male	48	47	49	1.10 (0.87–1.39)
Age (years)				
18–29	20	30	17	ref ^k^
30–44	25	32	23	1.29 (0.92–1.80)
45–59	24	22	25	**1.94 (1.37–2.74)**
60+	30	15	35	**4.00 (2.80–5.72)**
Education (attained)				
<High School	9	13	8	ref ^k^
High School	28	38	25	1.02 (0.68–1.54)
Some College	28	31	27	1.37 (0.92–2.05)
Bachelors or Higher	35	17	40	**3.70 (2.39–5.72)**
Race/Ethnicity				
White, non-Hispanic	63	64	63	ref ^k^
Black, non-Hispanic	12	15	11	**0.72 (0.54–0.96)**
Hispanic	17	16	17	1.11 (0.85–1.45)
Other, non-Hispanic	8	5	10	**2.09 (1.18–3.70)**
Region				
Northeast	17	14	18	ref ^k^
Midwest	21	22	20	0.70 (0.48–1.04)
South	38	42	37	**0.68 (0.48–0.96)**
West	24	22	25	0.86 (0.59–1.26)
Household income				
<$50K	30	43	26	ref ^k^
$50–85K	31	31	31	**1.65 (1.26–2.18)**
$85–150K	19	18	19	**1.74 (1.23–2.47)**
$150K+	20	8	23	**4.79 (3.19–7.20)**
Household size				
1	17	12	18	ref ^k^
2	35	27	38	0.95 (0.67–1.37)
3	19	22	18	**0.58 (0.39–0.87)**
4+	29	38	26	**0.47 (0.33–0.68)**
Number of children (ages 2–17)				
0	73	62	76	ref ^k^
1	11	14	10	**0.58 (0.40–0.84)**
2	11	14	10	**0.56 (0.39–0.80)**
3+	6	10	5	**0.40 (0.26–0.62)**
Political affiliation				
Republican	26	34	24	ref ^k^
Democrat	34	19	39	**2.91 (2.13–3.99)**
Independent	30	32	29	1.30 (0.96–1.77)
Something else	10	15	8	0.79 (0.54–1.15)
Metropolitan Statistical Area status (metro vs. non-metro)	87	81	88	**1.72 (1.22–2.42)**
Employment status (working vs. not working)	62	66	61	0.80 (0.63–1.02)
Physical health (good vs. not good)	84	84	84	0.96 (0.70–1.31)
Affirmative Responses to Survey Items ^e^				
*COVID-19 Disease*				
Have you ever had COVID-19?	18	30	15	**0.40 (0.30–0.53)**
How likely do you think it is that you will have COVID-19 over the next year?	20	24	18	**0.69 (0.52–0.93)**
When indoors in a crowded setting do you (or would you) wear a mask?	80	60	86	**4.20 (3.22–5.48)**
I am concerned that I or my family/friends will be exposed when others do not wear masks in public.	61	35	68	**3.91 (3.03–5.03)**
*COVID-19 Vaccine*				
How important do you think a COVID-19 vaccine is to stop the spread of infection in the US?	82	37	96	**39.10 (27.47–55.67)**
Are you worried that the COVID-19 vaccine is not safe for adults?	26	74	11	**0.04 (0.03–0.06)**
Have you discussed getting vaccinated with your healthcare provider?	36	28	39	**1.60 (1.24–2.06)**
Of those who have: the provider encouraged getting the vaccine.	70	32	79	**7.85 (4.89–12.59)**
*COVID-19 in Children*				
COVID-19 can be a serious disease for some children.	86	65	92	**6.04 (4.50–8.11)**
I am concerned about the safety of COVID-19 vaccine in children.	62	87	54	**0.18 (0.13–0.25)**
Vaccinating children against COVID-19 is important to end the pandemic and get back to normal.	71	22	85	**20.62 (15.47–27.49)**
It is better for children to develop immunity to COVID-19 by getting sick rather than by getting a shot.	29	67	17	**0.10 (0.08–0.13)**
COVID-19 in children is no worse than a cold or the flu.	32	62	23	**0.18 (0.14–0.23)**
*Vaccines Other than COVID-19*				
Had flu vaccination, past 12 months.	55	18	66	**9.00 (6.72–12.06)**
Of parents: Have you ever delayed having your child get a shot other than the flu for reasons other than illness or allergy?	16	30	9	**0.24 (0.14–0.41)**
Of parents: Have you ever decided not to have your child get a shot other than the flu for reasons other than illness or allergy?	11	21	6	**0.23 (0.13–0.44)**
Have you or anyone you know ever had a serious reaction to a vaccine?	8	24	4	**0.14 (0.10–0.20)**
*Healthcare and Science in General*				
Received high quality care from healthcare provider, past 12 months.	91	86	93	**2.13 (1.44–3.14)**
In general, would you say that you trust science?	90	77	95	**5.21 (3.70–7.34)**

Red text indicates survey items reflecting negative vaccine attitudes. ^a^ Column percentages (of total sample), weighted according to survey weights to achieve national representativeness. ^b^ Column percentages (of vaccinated/unvaccinated) (except for first row “All” which is a row percentage), weighted according to survey weights to achieve national representativeness. ^c^ Odds Ratio (95% Confidence Interval) of being vaccinated vs. unvaccinated for affirmative survey response vs. not; bold indicates statistical significance using the Pearson chi-square test at significance level of alpha = 5% (*p* < 0.05). ^d^ Construct scales combine scores for each relevant survey item (reversing negative items) and divide by maximum (e.g., 100 being complete trust and 0 being complete distrust); after dichotomizing at median, binary variable represents high vs. low score (e.g., 1 being high trust and 0 being low trust). ^e^ Likert scale response options (strongly agree, agree, disagree, strongly disagree, don’t know) dichotomized to agree/disagree (don’t know coded as disagree), results for agreement shown; other scale response options dichotomized to reflect affirmative/negative, results for affirmative shown. ^k^ Reference category for logistic regression of categorical variables.

**Table 3:** Frequency and Odds of Intention to Get COVID-19 Vaccine (3 groups) among Unvaccinated by Vaccine Attitudes, Trust in CDC and HDs, and Sociodemographic Characteristics. Numbers in the “Total” column indicate the percentage of the unvaccinated weighted sample providing the September 2021 survey response in each row. Numbers in the “Vaccine Intentions” columns indicate the percentage of those whose COVID-19 vaccine intentions match that of the column header who provided the survey response in each row. The numbers in the “OR (95%CI)” columns indicate the Odds Ratio comparing the different vaccine intentions matching that of the column header by the survey response in each row, boldface indicating statistical significance (*p* < 0.05).

Survey Items	Total Unvaccinated (%) ^a^	Vaccine Intentions, % ^b^			Likely vs. Unlikely	Uncertain vs. Unlikely
		Likely	Uncertain	Unlikely	OR (95%CI)^c^	OR (95%CI) ^c^
All (N = 523)	100	6	55	40		
Constructs ^d^						
Confidence in vaccines	16	54	18	8	**12.89 (4.71–35.31)**	**2.35 (1.17–4.73)**
Trust in the Centers for Disease Control and Prevention (CDC)	11	27	14	4	**8.96 (2.62–30.64)**	**3.98 (1.51–10.49)**
Trust in local and state health departments (HDs)	17	46	19	11	**7.06 (2.63–18.93)**	**2.02 (1.06–3.84)**
Sociodemographic Characteristics						
Gender						
Female	53	58	53	54	ref ^k^	ref ^k^
Male	47	42	47	46	0.83 (0.34–2.05)	1.05 (0.68–1.62)
Age (years)						
18–29	30	43	29	30	ref ^k^	ref ^k^
30–44	32	35	32	31	0.8 (0.27–2.37)	1.08 (0.60–1.95)
45–59	22	13	25	21	0.44 (0.13–1.45)	1.20 (0.65–2.24)
60+	15	9	14	19	0.35 (0.09–1.32)	0.75 (0.39–1.43)
Education (attained)						
<High School	13	9	13	14	ref ^k^	ref ^k^
High School	38	45	37	39	1.75 (0.36–8.39)	1.06 (0.52–2.17)
Some College	31	30	29	33	1.38 (0.27–7.02)	0.96 (0.47–1.95)
Bachelors or Higher	17	15	21	13	1.77 (0.29–10.76)	1.72 (0.76–3.88)
Race/Ethnicity						
White, non-Hispanic	64	41	63	69	ref ^k^	ref ^k^
Black, non-Hispanic	15	24	16	13	2.99 (0.99–9.01)	1.34 (0.77–2.35)
Hispanic	16	35	17	11	**5.17 (1.78–15.06)**	1.61 (0.95–2.74)
Other, non-Hispanic	5	0	4	6		0.76 (0.27–2.16)
Region						
Northeast	14	7	18	9	ref ^k^	ref ^k^
Midwest	22	8	21	27	0.42 (0.07–2.59)	**0.41 (0.19–0.87**)
South	42	57	42	40	1.89 (0.40–8.87)	0.54 (0.27–1.09)
West	22	28	19	24	1.56 (0.29–8.36)	**0.42 (0.19–0.90)**
Household income						
<$50K	43	42	46	40	ref ^k^	ref ^k^
$50–85K	31	31	30	33	0.87 (0.29–2.59)	0.79 (0.48–1.30)
$85–150K	18	23	16	19	1.17 (0.37–3.67)	0.74 (0.39–1.43)
$150K+	8	4	8	8	0.43 (0.05–3.67)	0.87 (0.40–1.92)
Household size						
1	12	12	11	14	ref ^k^	ref ^k^
2	27	16	30	26	0.71 (0.12–4.06)	1.45 (0.73–2.86)
3	22	10	24	20	0.55 (0.10–3.09)	1.46 (0.71–3.02)
4+	38	61	34	40	1.67 (0.37–7.52)	1.05 (0.55–2.01)
Number of children (ages 2–17)						
0	62	58	65	60	ref ^k^	ref ^k^
1	14	12	11	18	0.67 (0.18–2.55)	0.57 (0.29–1.10)
2	14	15	16	12	1.35 (0.31–5.81)	1.23 (0.64–2.37)
3+	10	16	8	10	1.54 (0.41–5.76)	0.75 (0.35–1.59)
Political affiliation						
Republican	34	27	30	40	ref ^k^	ref ^k^
Democrat	19	34	23	12	**4.31 (1.38–13.44)**	**2.62 (1.39–4.91)**
Independent	32	21	30	35	0.91 (0.26–3.21)	1.13 (0.65–1.96)
Something else	15	17	17	13	2 (0.44–9.15)	1.76 (0.92–3.35)
Metropolitan Statistical Area status (metro vs. non-metro)	81	91	78	84	1.82 (0.50–6.6)	0.67 (0.36–1.24)
Employment status (working vs. not working)	66	55	68	65	0.65 (0.26–1.61)	1.12 (0.71–1.77)
Physical health (good vs. not good)	84	92	81	88	1.56 (0.44–5.54)	0.58 (0.31–1.07)
Affirmative Responses to Survey Items ^e^						
*COVID-19 Disease*						
Have you ever had COVID-19?	30	29	27	37	0.69 (0.25–1.91)	0.63 (0.37–1.06)
How likely do you think it is that you will have COVID-19 over the next year?	24	54	27	16	**6.01 (2.24–16.18)**	**1.88 (1.04–3.39)**
When indoors in a crowded setting do you (or would you) wear a mask?	60	91	70	42	**13.87 (3.03–63.51)**	**3.18 (2.01–5.03)**
I am concerned that I or my family/friends will be exposed when others do not wear masks in public.	35	75	46	15	**17.50 (5.61–54.54)**	**4.87 (2.75–8.65)**
*COVID-19 Vaccine*						
How important do you think a COVID-19 vaccine is to stop the spread of infection in the US?	37	100	50	10		**8.82 (4.89–15.92)**
Are you worried that the COVID-19 vaccine is not safe for adults?	74	36	68	87	**0.08 (0.03–0.22)**	**0.32 (0.19–0.57)**
Have you discussed getting vaccinated with your healthcare provider?	28	27	29	27	1.02 (0.35–2.98)	1.14 (0.70–1.84)
Of those who have: the provider encouraged getting the vaccine.	32	39	30	31	1.39 (0.22–8.86)	0.94 (0.38–2.33)
*COVID-19 in Children*						
COVID-19 can be a serious disease for some children.	65	78	74	52	**3.37 (1.13–10.07)**	**2.67 (1.68–4.25)**
I am concerned about the safety of COVID-19 vaccine in children.	87	83	87	87	0.72 (0.22–2.38)	1.03 (0.53–2.00)
Vaccinating children against COVID-19 is important to end the pandemic and get back to normal.	22	89	26	7	**118.68 (35.67–394.86)**	**4.89 (2.39–10.00)**
It is better for children to develop immunity to COVID-19 by getting sick rather than by getting a shot.	67	35	61	80	**0.14 (0.05–0.37)**	**0.41 (0.25–0.68)**
COVID-19 in children is no worse than a cold or the flu.	62	39	53	78	**0.18 (0.07–0.48)**	**0.32 (0.19–0.52)**
*Vaccines Other than COVID-19*						
Had flu vaccination, past 12 months.	18	36	21	11	**4.63 (1.71–12.56)**	**2.23 (1.19–4.17)**
Of parents: Have you ever delayed having your child get a shot other than the flu for reasons other than illness or allergy?	30	23	28	32	0.63 (0.10–3.80)	0.82 (0.36–1.86)
Of parents: Have you ever decided not to have your child get a shot other than the flu for reasons other than illness or allergy?	21	6	23	23	0.22 (0.03–1.42)	1.02 (0.40–2.55)
Have you or anyone you know ever had a serious reaction to a vaccine?	24	7	21	30	**0.18 (0.06–0.55)**	0.62 (0.36–1.05)
*Healthcare and Science in General*						
Received high quality care from healthcare provider, past 12 months.	86	82	85	88	0.62 (0.15–2.52)	0.76 (0.36–1.60)
In general, would you say that you trust science?	77	97	77	73	**13.4 (2.84–63.1)**	1.29 (0.79–2.12)
*Of Unvaccinated: Mandates and Incentives ^i^*						
Of employed: If my employer required me to get the COVID-19 vaccine… I would get vaccinated. ^i^	14	49	16	5	**17.48 (4.05–75.47)**	**3.62 (1.16–11.31)**
Of those who would not:						
I would quit my job. ^i^	29	0	16	48		**0.21 (0.11–0.40)**
I would protest. ^i^	32	3	22	46	**0.04 (0.00–0.34)**	**0.32 (0.18–0.58)**
I would consider legal action. ^i^	43	5	29	63	**0.03 (0.01–0.17)**	**0.24 (0.13–0.43)**
I am not sure what I would do. ^i^	42	95	57	19	**83.13 (14.61–473.16)**	**5.89 (3.04–11.41)**
If I was offered a $25–$100 gift card for getting fully vaccinated… I would be more likely to get vaccinated. ^i^	5	25	7	<1	**161.67 (17.78–1469.68)**	**34.24 (4.36–269.14)**
If I was automatically enrolled in a lottery when I got fully vaccinated that made me eligible to win at least $100K… I would be more likely to get vaccinated. ^i^	6	19	7	2	**9.99 (2.09–47.87)**	2.94 (0.77–11.27)
Seeing fewer people wear masks in public makes me more likely to get vaccinated. ^i^	13	76	15	0		
*Of Unvaccinated: Knowledge and Decision-Making re: COVID-19 Vaccination ^i^*						
I am knowledgeable about COVID-19 vaccines for adults. ^i^	75	87	72	78	1.97 (0.47–8.30)	0.73 (0.43–1.22)
I still have many unanswered questions about COVID-19 vaccines for adults. ^i^	68	49	76	60	0.64 (0.26–1.55)	**2.13 (1.33–3.40)**
I still cannot decide whether getting the COVID-19 vaccine is best for me. ^i^	47	34	70	18	2.28 (0.87–6.00)	**10.46 (6.30–17.37)**
Talking with other people is important in helping me make up my mind about COVID-19 vaccination for myself. ^i^	35	44	43	22	**2.76 (1.08–7.03)**	**2.66 (1.63–4.33)**
*Of Unvaccinated: Specific Concerns and Other Reasons For Not Getting a COVID-19 Vaccine ^i^*						
How fast COVID-19 vaccines were developed and made available to the public. ^i^	59	52	57	61	0.68 (0.28–1.68)	0.86 (0.55–1.34)
COVID-19 vaccines are new. ^i^	80	73	79	81	0.63 (0.23–1.75)	0.85 (0.49–1.48)
The safety of COVID-19 vaccines has not been studied for a long enough period of time. ^i^	83	65	81	87	**0.27 (0.09–0.76)**	0.61 (0.32–1.17)
A lot of people who get the vaccine feel tired, achy and get headaches and fever. ^i^	74	78	74	72	1.38 (0.51–3.78)	1.07 (0.65–1.74)
Some people have bad allergic reactions to COVID-19 vaccines. ^i^	77	63	74	83	**0.34 (0.12–0.92)**	0.57 (0.33–1.00)
I am not sure the ingredients in COVID-19 vaccines are safe. ^i^	76	32	73	85	**0.08 (0.03–0.23)**	**0.47 (0.26–0.83)**
COVID-19 vaccines might change my genes or DNA (cause mutations). ^i^	33	9	24	48	**0.1 (0.03–0.31)**	**0.33 (0.21–0.53)**
COVID-19 vaccines might affect my fertility or ability to have children. ^i^	34	12	26	47	**0.15 (0.05–0.42)**	**0.40 (0.25–0.64)**
There were not enough people of my race/ethnicity who were a part of the vaccine studies. ^i^	16	14	17	15	0.96 (0.36–2.53)	1.19 (0.68–2.08)
They are experimenting on people with the COVID-19 vaccine. ^i^	69	36	61	84	**0.11 (0.04–0.29)**	**0.29 (0.17–0.50)**
The drug companies are making a lot of money off of COVID-19 vaccines. ^i^	70	55	69	75	0.42 (0.17–1.07)	0.76 (0.46–1.24)
Some COVID-19 vaccines are made from aborted fetuses. ^i^	23	8	17	34	**0.17 (0.05–0.55)**	**0.40 (0.24–0.68)**
I have a health condition that might make me at increased risk of having a bad reaction to the COVID-19 vaccine. ^i^	29	10	32	27	**0.32 (0.11–0.89)**	1.27 (0.79–2.04)
I have a health condition that would prevent the COVID-19 vaccine from being effective. ^i^	16	6	18	16	0.32 (0.08–1.31)	1.12 (0.63–1.98)
I am worried about severe vaccine side effects such as myocarditis (heart swelling), Guillain Barre Syndrome (paralysis), or severe blood clots. ^i^	74	59	73	79	0.38 (0.15–0.96)	0.71 (0.42–1.20)
Vaccine recommendations are influenced more by politics than by science. ^i^	83	44	79	92	**0.07 (0.02–0.18)**	**0.32 (0.16–0.63)**
I am worried about the safety of COVID-19 vaccines. ^i^	86	55	86	90	**0.13 (0.05–0.36)**	0.65 (0.33–1.28)
I do not trust how quickly the COVID-19 vaccine was developed. ^i^	86	59	86	90	**0.16 (0.06–0.45)**	0.67 (0.35–1.28)
I worry that I would have a reaction to the vaccine. ^i^	78	66	81	75	0.65 (0.25–1.67)	1.40 (0.83–2.34)
I worry about having to provide personal information (name, address, phone number, insurance card) to get the vaccine. ^i^	26	16	23	32	0.41 (0.13–1.35)	0.62 (0.38–1.02)
Those I trust (friends, family, or religious leaders) do not want to get the vaccine. ^i^	53	32	47	65	**0.24 (0.09–0.63)**	**0.48 (0.30–0.74)**
I have seen posts on social media that make me wary of the vaccine. ^i^	51	20	54	52	**0.23 (0.09–0.59)**	1.06 (0.69–1.64)
I want to wait to see what happens to others who are vaccinated. ^i^	73	61	81	64	0.89 (0.35–2.27)	**2.36 (1.45–3.82)**
I need more time to learn and think more about it. ^i^	59	31	80	34	0.88 (0.37–2.08)	**7.96 (4.90–12.94)**
Of pregnant women: I do not think the COVID-19 vaccine is safe for me or my baby. ^i^	39	48	41	31	2.02 (0.16–25.12)	1.56 (0.32–7.68)
*Of Unvaccinated: Reasons to Get COVID-19 Vaccination ^i^*						
COVID-19 vaccines are likely to protect me from the COVID-19 strains circulating. ^i^	24	81	34	3	**127.92 (36.05–453.88)**	**14.86 (6.09–36.24)**
COVID-19 vaccines are likely to protect me from new variants of COVID-19 that may appear in the future. ^i^	22	64	29	7	**24.48 (8.01–74.8)**	**5.56 (2.50–12.35)**
It’s important for me to get vaccinated so I don’t accidentally give COVID-19 to other people in my family. ^i^	21	90	29	1	**1037.84 (219.57–4905.59)**	**45.49 (12.93–160.10)**
It’s important for me to get vaccinated to help get my community back to normal. ^i^	17	84	20	2	**216.62 (56.06–836.96)**	**10.39 (3.78–28.52)**
The government is acting in my or my family’s best interest when it comes to COVID-19. ^i^	18	52	23	7	**13.32 (4.83–36.71)**	**3.77 (1.94–7.36)**
*Of Unvaccinated: Barriers to COVID-19 Vaccination ^i^*						
I cannot get transportation to where COVID-19 vaccines are being given. ^i^	5	1	7	2	0.65 (0.10–4.11)	3.08 (0.89–10.67)
The times when COVID-19 vaccines are being given conflict with my daily schedule. ^i^	12	47	12	6	**14.46 (4.49–46.54)**	2.37 (0.96–5.84)
I do not know how to register to get a vaccine appointment. ^i^	11	32	11	9	**4.79 (1.54–14.92)**	1.31 (0.61–2.83)
I know how to register to get a vaccine appointment but it is too difficult. ^i^	14	23	18	6	**4.25 (1.35–13.44)**	**3.11 (1.45–6.65)**
I cannot miss work to get vaccinated. ^i^	18	34	19	15	**2.91 (1.06–8.01)**	1.28 (0.69–2.36)
At least one of the above. ^i^	34	59	34	30	**3.40 (1.37–8.45)**	1.22 (0.75–1.97)

Red text indicates survey items reflecting negative vaccine attitudes. ^a^ Column percentages (of unvaccinated), weighted according to survey weights to achieve national representativeness. ^b^ Column percentages (of corresponding intention categories) (except for first row “All” which is a row percentage), weighted according to survey weights to achieve national representativeness. ^c^ Odds Ratio (95% Confidence Interval) of uncertainty vs. unlikeliness to receive COVID-19 vaccine for affirmative survey response vs. not; bold indicates statistical significance using the Pearson chi-square test at significance level of alpha = 5% (*p* < 0.05). ^d^ Construct scales combine scores for each relevant survey item (reversing negative items) and divide by maximum (e.g., 100 being complete trust and 0 being complete distrust); after dichotomizing at median, binary variable represents high vs. low score (e.g., 1 being high trust and 0 being low trust). ^e^ Likert scale response options (strongly agree, agree, disagree, strongly disagree, don’t know) dichotomized to agree/disagree (don’t know coded as disagree), results for agreement shown; other scale response options dichotomized to reflect affirmative/negative, results for affirmative shown. ^i^ asked only to unvaccinated respondents. ^k^ Reference category for logistic regression of categorical variables.

**Table 4:** Race/Ethnicity by Vaccine Attitudes, Trust in CDC and HDs, and Sociodemographic Characteristics. Numbers in the “Total” column indicate the percentage of the total weighted sample providing the September 2021 survey response in each row. Numbers in the “Race/Ethnicity” columns indicate the percentage of those whose race/ethnicity match that of the column header who provided the survey response in each row. The numbers in the final column indicate the *p*-value of this association, boldface indicating statistical significance (*p* < 0.05).

Survey Items	Total (%) ^a^	Race/Ethnicity (%) ^b^				*p*-Value ^c^
		White, Non-Hispanic	Black, Non-Hispanic	Hispanic	Other, Non-Hispanic	
All (N = 2546)	100	63	12	17	8	
Constructs ^d^						
Confidence in vaccines	65	67	51	62	70	**<0.01**
Trust in the Centers for Disease Control and Prevention (CDC)	47	46	46	49	47	0.85
Trust in local and state health departments (HDs)	46	45	48	47	46	0.81
Sociodemographic Characteristics						
Gender						0.33
Female	52	52	55	50	46	
Male	48	48	45	50	54	
Age (years)						**<0.01**
18–29	20	17	24	27	26	
30–44	25	23	27	32	31	
45–59	24	25	24	24	20	
60+	30	36	25	17	23	
Education (attained)						**<0.01**
<High School	9	5	11	24	11	
High School	28	28	33	31	10	
Some College	28	29	30	26	20	
Bachelors or Higher	35	38	26	19	59	
Region						**<0.01**
Northeast	17	18	16	14	22	
Midwest	21	26	17	9	13	
South	38	36	58	38	26	
West	24	21	9	39	39	
Household income						**<0.01**
<$50K	30	28	44	35	21	
$50–85K	31	31	31	34	25	
$85–150K	19	19	14	18	20	
$150K+	20	22	10	13	33	
Household size						**<0.01**
1	17	17	25	11	16	
2	35	40	31	25	29	
3	19	19	16	20	21	
4+	29	25	28	45	33	
Number of children (ages 2–17)						**<0.01**
0	73	75	74	61	74	
1	11	9	12	14	14	
2	11	10	9	15	7	
3+	6	5	5	10	5	
Political affiliation						**<0.01**
Republican	26	34	3	15	16	
Democrat	34	27	63	42	32	
Independent	30	30	22	27	43	
Something else	10	9	11	16	9	
Metropolitan Statistical Area status (metro vs. non-metro)	87	83	91	94	95	**<0.01**
Employment status (working vs. not working)	62	60	60	67	69	0.06
Physical health (good vs. not good)	84	84	81	83	90	0.16
Affirmative Responses to Survey Items ^e^						
*COVID-19 Disease*						
Have you ever had COVID-19?	18	17	17	22	17	0.24
How likely do you think it is that you will have COVID-19 over the next year?	20	18	17	27	20	**0.01**
When indoors in a crowded setting do you (or would you) wear a mask?	80	75	90	88	89	**<0.01**
I am concerned that I or my family/friends will be exposed when others do not wear masks in public.	61	53	78	74	76	**<0.01**
*COVID-19 Vaccine*						
Have you received a COVID-19 vaccine?	77	77	70	78	87	**<0.01**
Of those who have not, COVID-19 vaccine intentions:						**0.03**
Likely	6	4	9	13	0	
Uncertain	55	54	57	58	49	
Unlikely	40	43	34	29	51	
How important do you think a COVID-19 vaccine is to stop the spread of infection in the US?	82	80	85	88	88	**<0.01**
Are you worried that the COVID-19 vaccine is not safe for adults?	26	27	26	24	21	0.42
Have you discussed getting vaccinated with your healthcare provider?	36	38	39	34	27	**0.04**
Of those who have: the provider encouraged getting the vaccine.	70	73	64	70	57	0.11
*COVID-19 in Children*						
COVID-19 can be a serious disease for some children.	86	84	90	89	91	**0.01**
I am concerned about the safety of COVID-19 vaccine in children.	62	59	70	67	60	**<0.01**
Vaccinating children against COVID-19 is important to end the pandemic and get back to normal.	71	66	75	78	81	**<0.01**
It is better for children to develop immunity to COVID-19 by getting sick rather than by getting a shot.	29	31	26	27	20	**0.02**
COVID-19 in children is no worse than a cold or the flu.	32	35	26	29	18	**<0.01**
*Vaccines Other than COVID-19*						
Had flu vaccination, past 12 months.	55	58	44	49	55	**<0.01**
Of parents: Have you ever delayed having your child get a shot other than the flu for reasons other than illness or allergy?	16	18	12	12	18	0.34
Of parents: Have you ever decided not to have your child get a shot other than the flu for reasons other than illness or allergy?	11	10	16	8	18	0.19
Have you or anyone you know ever had a serious reaction to a vaccine?	8	9	7	7	5	0.14
*Healthcare and Science in General*						
Received high quality care from healthcare provider, past 12 months.	91	92	89	87	92	0.06
In general, would you say that you trust science?	90	91	87	92	89	0.24
*Among Vaccinated: Boosters ^j^*						
If the CDC were to recommend a booster dose so your body can continue to protect you against COVID-19, how likely are you to get one? ^j^	87	86	79	90	93	**0.01**
*Of Unvaccinated: Mandates and Incentives ^i^*						
Of employed: If my employer required me to get the COVID-19 vaccine… I would get vaccinated. ^i^	14	13	17	15	13	0.85
Of those who would not:						
I would quit my job. ^i^	29	31	30	25	18	0.63
I would protest. ^i^	32	35	15	33	29	0.10
I would consider legal action. ^i^	43	47	23	39	53	**0.03**
I am not sure what I would do. ^i^	42	40	49	43	44	0.80
If I was offered a $25-$100 gift card for getting fully vaccinated… I would be more likely to get vaccinated. ^i^	5	6	2	6	4	0.44
If I was automatically enrolled in a lottery when I got fully vaccinated that made me eligible to win at least $100K… I would be more likely to get vaccinated. ^i^	6	5	7	9	0	0.33
Seeing fewer people wear masks in public makes me more likely to get vaccinated. ^i^	13	8	24	21	12	**0.01**
*Of Unvaccinated: Knowledge and Decision-Making re: COVID-19 Vaccination ^i^*						
I am knowledgeable about COVID-19 vaccines for adults. ^i^	75	76	69	76	77	0.70
I still have many unanswered questions about COVID-19 vaccines for adults. ^i^	68	68	68	71	63	0.92
I still cannot decide whether getting the COVID-19 vaccine is best for me. ^i^	47	46	50	50	52	0.82
Talking with other people is important in helping me make up my mind about COVID-19 vaccination for myself. ^i^	35	33	41	35	52	0.25
*Of Unvaccinated: Specific Concerns and Other Reasons For Not Getting a COVID-19 Vaccine ^i^*						
How fast COVID-19 vaccines were developed and made available to the public. ^i^	59	62	45	61	55	0.09
COVID-19 vaccines are new. ^i^	80	85	66	74	71	**0.01**
The safety of COVID-19 vaccines has not been studied for a long enough period of time. ^i^	83	87	69	80	78	**0.02**
A lot of people who get the vaccine feel tired, achy and get headaches and fever. ^i^	74	75	59	82	68	**0.02**
Some people have bad allergic reactions to COVID-19 vaccines. ^i^	77	80	71	71	78	0.32
I am not sure the ingredients in COVID-19 vaccines are safe. ^i^	76	80	62	69	78	**0.02**
COVID-19 vaccines might change my genes or DNA (cause mutations). ^i^	33	34	33	24	35	0.37
COVID-19 vaccines might affect my fertility or ability to have children. ^i^	34	34	28	36	42	0.61
There were not enough people of my race/ethnicity who were a part of the vaccine studies. ^i^	16	10	26	27	31	**<0.01**
They are experimenting on people with the COVID-19 vaccine. ^i^	69	72	54	68	76	0.05
The drug companies are making a lot of money off of COVID-19 vaccines. ^i^	70	75	59	63	70	0.06
Some COVID-19 vaccines are made from aborted fetuses. ^i^	23	23	22	25	24	0.96
I have a health condition that might make me at increased risk of having a bad reaction to the COVID-19 vaccine. ^i^	29	27	30	25	52	0.10
I have a health condition that would prevent the COVID-19 vaccine from being effective. ^i^	16	15	15	18	35	0.10
I am worried about severe vaccine side effects such as myocarditis (heart swelling), Guillain Barre Syndrome (paralysis), or severe blood clots. ^i^	74	76	62	83	70	**0.04**
Vaccine recommendations are influenced more by politics than by science. ^i^	83	88	66	75	84	**<0.01**
I am worried about the safety of COVID-19 vaccines. ^i^	86	86	84	84	89	0.88
I do not trust how quickly the COVID-19 vaccine was developed. ^i^	86	89	76	85	81	0.07
I worry that I would have a reaction to the vaccine. ^i^	78	77	81	79	77	0.90
I worry about having to provide personal information (name, address, phone number, insurance card) to get the vaccine. ^i^	26	25	28	34	15	0.37
Those I trust (friends, family, or religious leaders) do not want to get the vaccine. ^i^	53	60	42	38	58	**<0.01**
I have seen posts on social media that make me wary of the vaccine. ^i^	51	50	52	55	52	0.89
I want to wait to see what happens to others who are vaccinated. ^i^	73	74	74	75	59	0.48
I need more time to learn and think more about it. ^i^	59	57	71	58	53	0.19
Of pregnant women: I do not think the COVID-19 vaccine is safe for me or my baby. ^i^	39	58	25	16	0	0.12
*Of Unvaccinated: Reasons to Get COVID-19 Vaccination ^i^*						
COVID-19 vaccines are likely to protect me from the COVID-19 strains circulating. ^i^	24	22	33	32	4	**<0.01**
COVID-19 vaccines are likely to protect me from new variants of COVID-19 that may appear in the future. ^i^	22	17	38	32	4	**<0.01**
It’s important for me to get vaccinated so I don’t accidentally give COVID-19 to other people in my family. ^i^	21	16	37	29	17	**0.01**
It’s important for me to get vaccinated to help get my community back to normal. ^i^	17	12	29	28	0	**<0.01**
The government is acting in my or my family’s best interest when it comes to COVID-19. ^i^	18	14	28	33	4	**<0.01**
*Of Unvaccinated: Barriers to COVID-19 Vaccination ^i^*						
I cannot get transportation to where COVID-19 vaccines are being given. ^i^	5	4	5	7	0	0.52
The times when COVID-19 vaccines are being given conflict with my daily schedule. ^i^	12	10	18	15	9	0.34
I do not know how to register to get a vaccine appointment. ^i^	11	10	17	13	0	0.17
I know how to register to get a vaccine appointment but it is too difficult. ^i^	14	10	25	16	14	**0.03**
I cannot miss work to get vaccinated. ^i^	18	16	19	25	18	0.46
At least one of the above. ^i^	34	29	46	38	41	0.06

Red text indicates survey items reflecting negative vaccine attitudes. ^a^ Column percentages (of total sample), weighted according to survey weights to achieve national representativeness. ^b^ Column percentages (of race/ethnicity) (except for first row “All” which is a row percentage), weighted according to survey weights to achieve national representativeness. ^c^ using the Pearson chi-square test at significance level of alpha = 5%; bold indicates statistical significance (*p* < 0.05). ^d^ Construct scales combine scores for each relevant survey item (reversing negative items) and divide by maximum (e.g., 100 being complete trust and 0 being complete distrust); after dichotomizing at median, binary variable represents high vs. low score (e.g., 1 being high trust and 0 being low trust). ^e^ Likert scale response options (strongly agree, agree, disagree, strongly disagree, don’t know) dichotomized to agree/disagree (don’t know coded as disagree), results for agreement shown; other scale response options dichotomized to reflect affirmative/negative, results for affirmative shown. ^i^ asked only to unvaccinated respondents. ^j^ asked only to vaccinated respondents.

**Table 5:** Changes in Vaccine Attitudes and Trust in CDC and HDs Between December 2020 and September 2021 by Political Affiliation. Numbers in the “Dec2020%” column indicate the percentage of the total weighted sample providing the December 2020 survey response in each row. Numbers in the “Sept2021%” column indicate the percentage of the total weighted sample providing the September 2021 survey response in each row. Numbers in the “%dif” column indicates the change between these two surveys. The numbers in the next column indicate the *p*-value of this difference, boldface indicating statistical significance (*p* < 0.05). Numbers in the “Republican” and “Democrat” columns indicate the percentage of the total weighted samples in December 2020 or September 2021 providing the survey response in each row who were Republican and Democrat, respectively.

Survey Items	Dec2020% ^b^ (*n* = 2525)	Sept2021% ^b^ (*n* = 2546)	%dif	*p*-Value ^c^	Dec2020% ^b^ Republican	Sept2021% ^b^ Republican	%dif	*p*-Value ^c^	Dec2020% ^b^ Democrat	Sept2021% ^b^ Democrat	%dif	*p*-Value ^c^
Constructs ^d^												
Confidence in vaccines	54.5	61.9	7.4	<0.01	51.6	55.2	3.6	0.01	59.0	71.0	12.0	**<0.01**
Trust in the Centers for Disease Control and Prevention (CDC)	60.1	59.4	−0.7	0.33	55.3	53.1	−2.2	0.07	66.2	69.1	2.9	**<0.01**
Trust in local and state health departments (HDs)	57.0	56.1	−0.9	0.20	54.0	52.8	−1.2	0.28	62.1	63.2	1.1	0.21
Affirmative Responses to Survey Items ^e^												
Have you ever had COVID-19?	4.5	18.0	13.5	<0.01	4.6	18.4	13.8	<0.01	5.4	17.2	11.8	**<0.01**
How likely do you think it is that you will have COVID-19 over the next year?	37.3	19.6	−17.7	<0.01	38.3	19.8	−18.5	<0.01	39.9	21.2	−18.7	**<0.01**
How important do you think a COVID-19 vaccine is to stop the spread of infection in the US?	88.2	82.4	−5.8	<0.01	84.8	73.9	−10.9	<0.01	95.3	94.8	−0.5	0.66

^b^ Column percentages (of corresponding sample), weighted according to survey weights to achieve national representativeness. ^c^ significance level of alpha = 5%; bold indicates statistical significance (*p* < 0.05). ^d^ Construct scales combine scores for each relevant survey item (reversing negative items) and divide by maximum (e.g., 100 being complete trust and 0 being complete distrust). ^e^ Scale response options dichotomized to reflect affirmative/negative, results for affirmative shown.

## Data Availability

Please see Supplemental Materials section.

## Supplementary Materials

The following supporting information can be downloaded at: https://www.mdpi.com/article/10.3390/jcm11133734/s1, Table S1: Frequency and Odds of COVID-19 Disease by Vaccine Attitudes, Trust in CDC and HDs, and Sociodemographic Characteristics; Table S2: Frequency and Odds of Hesitation Before Getting COVID-19 Vaccine among Vaccinated by Vaccine Attitudes, Trust in CDC and HDs, and Sociodemographic Characteristics; Table S3: Frequency and Odds of COVID-19 Vaccination among Hesitant by Vaccine Attitudes, Trust in CDC and HDs, and Sociodemographic Characteristics; Table S4: Gender by Vaccine Attitudes, Trust in CDC and HDs, and Sociodemographic Characteristics; Table S5: Political Affiliation by Vaccine Attitudes, Trust in CDC and HDs, and Sociodemographic Characteristics; Table S6: Age by Vaccine Attitudes, Trust in CDC and HDs, and Sociodemographic Characteristics; Table S7: Income by Vaccine Attitudes, Trust in CDC and HDs, and Sociodemographic Characteristics; Table S8: Employment Status by Vaccine Attitudes, Trust in CDC and HDs, and Sociodemographic Characteristics; Table S9: Metropolitan Statistical Area Status by Vaccine Attitudes, Trust in CDC and HDs, and Sociodemographic Characteristics; Table S10: Physical Health by Vaccine Attitudes, Trust in CDC and HDs, and Sociodemographic Characteristics; Table S11: Frequency and Odds of COVID-19 Vaccination by Political Activities and Support, Sources of Health Information, and Reasons For Not Getting the Flu Vaccine; Table S12: Frequency and Odds of Intention to Get COVID-19 Vaccine (3 groups) among Unvaccinated by Political Activities and Support, Sources of Health Information, and Reasons For Not Getting the Flu Vaccine; Table S13: Race/Ethnicity by Political Activities and Support, Sources of Health Information, and Reasons For Not Getting the Flu Vaccine; Table S14: Race/Ethnicity among Unvaccinated and Uncertain by Vaccine Attitudes and Trust in CDC and HDs; Table S15: Composition and Properties of Construct Scales.
