# Supplementary material for: COVID-19 Vaccination Status, Attitudes, and Values among US Adults in September 2021
Source: J Clin Med. 2022 Jun 28;11(13):3734. doi: 10.3390/jcm11133734 (PMC9267733; doi:10.3390/jcm11133734)
Supplement: Supplementary file 1 [file jcm-11-03734-s001.zip › Table S10.pdf]

Table S10. Physical Health by Vaccine Attitudes, Trust in CDC and HDs, and Sociodemographic Characteristics

Numbers in the "Total" column indicate the percentage of the total weighted sample providing the September 2021 survey response in each row. Numbers in the "Physical Health" columns indicate the percentage of those whose physical health match that of the column header who provided the survey response in each row. The numbers in the final column indicate the p-value of this association, boldface indicating statistical significance (p<0.05).

Survey Items	Total (%)[a]	Physical Health (%)[b]		p-value[c]
		Not Good	Good	
**All**	100	16	84	
**Constructs** [d]				
Confidence in vaccines	65	63	65	0.49
Trust in the Centers for Disease Control and Prevention (CDC)	47	45	47	0.42
Trust in local and state health departments (HDs)	46	41	47	0.07
**Sociodemographic Characteristics**				
Gender				0.20
Female	52	55	51	
Male	48	45	49	
Age (years)				0.24
18-29	20	16	21	
30-44	25	27	25	
45-59	24	25	24	
60+	30	33	30	
Education (attained)				**<0.01**
<High School	9	18	8	

High School	28	38	26	
Some College	28	21	29	
Bachelors or Higher	35	24	37	
Race/Ethnicity				0.16
White, non-Hispanic	63	63	63	
Black, non-Hispanic	12	14	11	
Hispanic	17	5	9	
Other, non-Hispanic	8	18	16	
Region				0.90
Northeast	17	19	17	
Midwest	21	20	21	
South	38	38	38	
West	24	23	24	
Metropolitan Statistical Area status (metro vs non-metro)	87	85	87	0.32
Employment status (working vs not working)	62	43	66	**<0.01**
Household income				**<0.01**
<$50k	30	45	27	
$50-85k	31	26	32	
$85-150k	19	16	19	
$150k+	20	12	21	
Household size				0.28
1	17	20	16	
2	35	32	36	

3	19	19	19	
4+	29	28	29	
Number of children (ages 2-17)				0.57
0	73	76	72	
1	11	10	11	
2	11	8	11	
3+	6	6	6	
Political affiliation				**<0.01**
Republican	26	18	27	
Democrat	34	43	33	
Independent	30	26	30	
Something else	10	13	10	
**Affirmative Responses to Survey Items** [e]				
COVID-19 Disease				
Have you ever had COVID-19?	18	18	18	0.98
How likely do you think it is that you will have COVID-19 over the next year?	20	23	19	0.15
When indoors in a crowded setting do you (or would you) wear a mask?	80	84	80	0.11
I am concerned that I or my family/friends will be exposed when others do not wear masks in public.	61	69	59	**<0.01**
COVID-19 Vaccine				
Have you received a COVID-19 vaccine?	77	78	77	0.79
How important do you think a COVID-19 vaccine is to stop the	82	84	82	0.47

spread of infection in the US?				
Are you worried that the COVID-19 vaccine is not safe for adults?	26	27	25	0.53
Have you discussed getting vaccinated with your healthcare provider?	36	42	35	**0.02**
Of those who have: the provider encouraged getting the vaccine.	70	65	71	0.16
COVID-19 in Children				
COVID-19 can be a serious disease for some children.	86	87	86	0.51
I am concerned about the safety of COVID-19 vaccine in children.	62	64	61	0.43
Vaccinating children against COVID-19 is important to end the pandemic and get back to normal.	71	73	70	0.38
It is better for children to develop immunity to COVID-19 by getting sick rather than by getting a shot.	29	26	29	0.32
COVID-19 in children is no worse than a cold or the flu.	32	24	33	**<0.01**
Vaccines Other than COVID-19				
Had flu vaccination, past 12 months.	55	55	55	0.89
Of parents: Have you ever delayed having your child get a shot other than the flu for reasons other than illness or allergy?	16	16	16	0.95
Of parents: Have you ever decided not to have your child get a shot other than the flu for reasons other than illness or allergy?	11	14	10	0.40
Have you or anyone you know ever had a serious reaction to a vaccine?	8	8	9	0.66
Healthcare and Science in General				
Received high quality care from healthcare provider, past 12 months.	91	82	93	**<0.01**
In general, would you say that you trust science?	90	87	91	**0.04**
Among Vaccinated: Boosters[j]				
If the CDC were to recommend a booster dose so your body can	87	83	87	0.10

continue to protect you against COVID-19, how likely are you to get one? [j]				
Of Unvaccinated: Mandates and Incentives [i]				
Of employed: If my employer required me to get the COVID-19 vaccine... I would get vaccinated. [i]	14	17	13	0.55
Of those who would not:				
I would quit my job. [i]	29	13	32	**0.04**
I would protest. [i]	32	19	34	0.09
I would consider legal action. [i]	43	42	43	0.93
I am not sure what I would do. [i]	42	46	41	0.58
If I was offered a $25-$100 gift card for getting fully vaccinated... I would be more likely to get vaccinated. [i]	5	11	4	**0.03**
If I was automatically enrolled in a lottery when I got fully vaccinated that made me eligible to win at least $100K... I would be more likely to get vaccinated. [i]	6	7	5	0.72
Seeing fewer people wear masks in public makes me more likely to get vaccinated. [i]	13	22	11	**0.02**
Of Unvaccinated: Knowledge and Decision-Making re: COVID-19 Vaccination [i]				
I am knowledgeable about COVID-19 vaccines for adults. [i]	75	58	78	**<0.01**
I still have many unanswered questions about COVID-19 vaccines for adults. [i]	68	66	69	0.68
I still cannot decide whether getting the COVID-19 vaccine is best for me. [i]	47	45	48	0.74
Talking with other people is important in helping me make up my mind about COVID-19 vaccination for myself. [i]	35	31	36	0.45

Of Unvaccinated: Specific Concerns and Other Reasons For Not Getting a COVID-19 Vaccine [i]				
How fast COVID-19 vaccines were developed and made available to the public. [i]	59	45	61	**0.02**
COVID-19 vaccines are new. [i]	80	73	81	0.16
The safety of COVID-19 vaccines has not been studied for a long enough period of time. [i]	83	75	84	0.13
A lot of people who get the vaccine feel tired, achy and get headaches and fever. [i]	74	67	75	0.20
Some people have bad allergic reactions to COVID-19 vaccines. [i]	77	67	79	**0.04**
I am not sure the ingredients in COVID-19 vaccines are safe. [i]	76	72	76	0.53
COVID-19 vaccines might change my genes or DNA (cause mutations). [i]	33	27	33	0.36
COVID-19 vaccines might affect my fertility or ability to have children. [i]	34	30	35	0.47
There were not enough people of my race/ethnicity who were a part of the vaccine studies. [i]	16	18	16	0.69
They are experimenting on people with the COVID-19 vaccine. [i]	69	62	70	0.21
The drug companies are making a lot of money off of COVID-19 vaccines. [i]	70	60	72	0.07
Some COVID-19 vaccines are made from aborted fetuses. [i]	23	16	24	0.16
I have a health condition that might make me at increased risk of having a bad reaction to the COVID-19 vaccine. [i]	29	44	26	**<0.01**
I have a health condition that would prevent the COVID-19 vaccine from being effective. [i]	16	22	15	0.17
I am worried about severe vaccine side effects such as myocarditis (heart swelling), Guillain Barre Syndrome (paralysis), or severe blood clots. [i]	74	66	76	0.12

Vaccine recommendations are influenced more by politics than by science. [i]	83	72	85	**0.02**
I am worried about the safety of COVID-19 vaccines. [i]	86	84	86	0.61
I do not trust how quickly the COVID-19 vaccine was developed. [i]	86	85	86	0.89
I worry that I would have a reaction to the vaccine. [i]	78	80	78	0.72
I worry about having to provide personal information (name, address, phone number, insurance card) to get the vaccine. [i]	26	28	26	0.77
Those I trust (friends, family, or religious leaders) do not want to get the vaccine. [i]	53	44	55	0.12
I have seen posts on social media that make me wary of the vaccine. [i]	51	50	51	0.89
I want to wait to see what happens to others who are vaccinated. [i]	73	67	74	0.24
I need more time to learn and think more about it. [i]	59	61	59	0.84
Of pregnant women: I do not think the COVID-19 vaccine is safe for me or my baby. [i]	39	39	39	0.99
Of Unvaccinated: Reasons to Get COVID-19 Vaccination [i]				
COVID-19 vaccines are likely to protect me from the COVID-19 strains circulating. [i]	24	23	24	0.86
COVID-19 vaccines are likely to protect me from new variants of COVID-19 that may appear in the future. [i]	22	31	21	0.11
It's important for me to get vaccinated so I don't accidentally give COVID-19 to other people in my family. [i]	21	25	21	0.55
It's important for me to get vaccinated to help get my community back to normal. [i]	17	20	16	0.41
The government is acting in my or my family's best interest when it comes to COVID-19. [i]	18	25	17	0.17
Of Unvaccinated: Barriers to COVID-19 Vaccination [i]				

I cannot get transportation to where COVID-19 vaccines are being given. [i]	5	5	5	0.91
The times when COVID-19 vaccines are being given conflict with my daily schedule. [i]	12	14	11	0.50
I do not know how to register to get a vaccine appointment. [i]	11	11	11	0.93
I know how to register to get a vaccine appointment but it is too difficult. [i]	14	12	14	0.69
I cannot miss work to get vaccinated. [i]	18	23	17	0.33
At least one of the above. [i]	34	42	32	0.15
Political Activities and Support				
People may be involved in civic and political activities. In the past 12 months, have you...				
Attended a political protest or rally	7	4	7	**0.04**
Contacted a government official	15	16	15	0.65
Volunteered or worked for a Presidential campaign	3	2	3	0.23
Volunteered or worked for a political candidate other than a Presidential campaign	2	1	3	**0.04**
Volunteered or worked for a political party, issue, or cause	4	2	4	0.12
Served on a committee for a civic, non-profit or community organization	5	3	6	0.05
Written a letter or email to a newspaper/magazine or called a live radio or TV show	4	6	4	0.10
Commented about politics on a message board or internet site	19	22	19	0.18
Shared your opinion about a town or community issue at a public meeting	4	5	4	0.58
Held a publicly elected office	0	0	0	0.27
Signed a petition	26	25	26	0.77

Ran for a publicly elected office	0	0	0	0.40
None of these	60	58	61	0.42
Do you identify with or actively support any of the following political movements?				
Tea Party (Taxed Enough Already)	6	6	6	0.68
Environmental Rights	20	20	20	0.92
Women's Rights/ Me Too	23	27	23	0.19
Racial Equality	26	28	26	0.53
Right to Life	15	11	15	0.06
Peace/Anti-War	11	14	11	0.21
Lesbian, Gay, Bisexual, Transgender, Queer (LGBTQ) Rights	19	23	19	0.11
Indivisible	2	2	2	0.55
Black Lives Matter	25	28	24	0.23
Men's Rights	4	5	4	0.47
Alt-right	1	0	1	0.39
Boogaloo movement	0	1	0	0.29
Antifa	2	2	2	0.94
QAnon	1	0	1	**<0.01**
Anti-gun violence	16	15	16	0.65
None of these	53	54	52	0.69
Do you identify with or actively support any of the following organizations?				
National Rifle Association (NRA)	13	10	14	0.05
Heritage Foundation	3	3	3	0.79
Planned Parenthood	19	18	19	0.64
National Right to Life Committee	5	4	5	0.20
Greenpeace	6	7	6	0.81
Sierra Club	8	7	8	0.59

Amnesty International	6	5	6	0.60
National Education Association Foundation	6	9	6	0.08
American Civil Liberties Union (ACLU)	12	11	12	0.64
Americans for Prosperity	1	1	1	0.79
MoveOn.org	7	6	7	0.85
The NAACP/National Association for the Advancement of Colored People	12	13	12	0.72
American Red Cross	23	19	24	0.06
Chamber of Commerce	3	4	3	0.21
Freedom Caucus	2	1	2	0.22
None of these	53	58	52	0.08
Sources of Health Information				
Which of the following sources have you used to look for health and wellness related information or education in the past 12 months?				
Doctor	60	61	60	0.84
Pharmacist	21	27	20	**0.01**
Nurse, nurse practitioner or physician's assistant	29	33	28	0.07
Relative, friend or co-worker	23	22	23	0.68
Someone you know who has a particular medical condition	8	10	8	0.31
Disease-related association or society	5	5	6	0.53
Patient support group or foundation	2	2	2	0.89
Educational forum at a local clinic, hospital, community center or other location	3	3	4	0.64
Pharmaceutical company	1	2	1	0.65
Health insurance company	7	6	7	0.52
Newspapers or magazines	7	7	6	0.49
Television	6	6	6	0.88

The internet	48	49	48	0.83
Social Media (such as Facebook, Twitter)	6	7	5	0.27
Healthcare app for smartphone or tablet	6	7	6	0.45
Have not looked for information in the past 12 months	21	20	21	0.79
Barriers, Specific Concerns and Other Reasons For Not Getting the Flu Vaccine				
Of those who did not get a flu shot this past year: this is because… [i]				
The flu is not a serious illness	9	9	10	0.72
I'm healthy	22	6	25	**<0.01**
I just didn't think about it	22	21	23	0.64
I didn't know where to get it	1	3	1	**0.01**
I didn't have health insurance	3	6	3	0.09
I didn't have time	5	7	5	0.32
I don't believe in vaccines	6	7	6	0.82
I'm afraid of the side effects	11	14	10	0.24
I'm afraid of needles	4	5	4	0.96
I prefer alternative (homeopathic) medicine to vaccines	11	5	12	**0.04**
I have never had the flu	13	11	13	0.43
The vaccine will make me sick with the flu	9	14	9	0.08
I got a flu shot the year before so I didn't need it	2	3	2	0.55
Another reason	27	29	27	0.56

Red text indicates survey items reflecting negative vaccine attitudes

[a] Column percentages (of total sample), weighted according to survey weights to achieve national representativeness

[b] Column percentages (of physical health categories) (except for first row "All" which is a row percentage), weighted according to survey weights to achieve national representativeness

[c] using the Pearson chi-square test at significance level of alpha=5%; bold indicates statistical significance ($p<0.05$)

[d] Construct scales combine scores for each relevant survey item (reversing negative items) and divide by maximum (e.g., 100 being complete trust and 0 being complete distrust); after dichotomizing at median, binary variable represents high vs low score (e.g., 1 being high trust and 0 being low trust)

[e] Likert scale response options (strongly agree, agree, disagree, strongly disagree, don't know) dichotomized to agree/disagree (don't know coded as disagree), results for agreement shown; other scale response options dichotomized to reflect affirmative/negative, results for affirmative shown

[i] asked only to unvaccinated respondents

[j] asked only to vaccinated respondents
