# Supplementary material for: COVID-19 Vaccination Status, Attitudes, and Values among US Adults in September 2021
Source: J Clin Med. 2022 Jun 28;11(13):3734. doi: 10.3390/jcm11133734 (PMC9267733; doi:10.3390/jcm11133734)
Supplement: Supplementary file 1 [file jcm-11-03734-s001.zip › Table S11.pdf]

Table S11. Frequency and Odds of COVID-19 Vaccination by Political Activities and Support, Sources of Health Information, and Reasons For Not Getting the Flu Vaccine

Numbers in the "Total" column indicate the percentage of the total weighted sample providing the September 2021 survey response in each row. Numbers in the "COVID-19 Vaccination" columns indicate the percentage of those whose COVID-19 vaccination status match that of the column header who provided the survey response in each row. The numbers in the "OR (95%CI)" column indicate the Odds Ratio of being vaccinated vs unvaccinated by the survey response in each row. The numbers in the final column indicate the p-value of this association, boldface indicating statistical significance (p<0.05).

Survey Items	Total (%)[a]	COVID-19 Vaccination, %[b] Unvaccinated	Vaccinated	OR (95%CI)[j]	p-value[c]
**All**	100	23	77		
**Affirmative Responses to Survey Items** [e]					
Political Activities and Support					
People may be involved in civic and political activities. In the past 12 months, have you...					
Attended a political protest or rally	7	4	7	1.73 (0.99-3.01)	0.05
Contacted a government official	15	13	16	1.35 (0.95-1.92)	0.09
Volunteered or worked for a Presidential campaign	3	2	3	1.97 (0.79-4.89)	0.14
Volunteered or worked for a political candidate other than a Presidential campaign	2	1	3	2.18 (0.74-6.44)	0.15
Volunteered or worked for a political party, issue, or cause	4	2	4	2.16 (1.03-4.54)	**0.04**
Served on a committee for a civic, non-profit or community organization	5	3	6	1.75 (0.95-3.22)	0.07
Written a letter or email to a	4	5	4	0.80 (0.45-1.44)	0.46

newspaper/magazine or called a live radio or TV show					
Commented about politics on a message board or internet site	19	18	20	1.12 (0.83-1.51)	0.48
Shared your opinion about a town or community issue at a public meeting	4	4	4	1.28 (0.66-2.46)	0.46
Held a publicly elected office	0	0	0	1.56 (0.18-13.21)	0.68
Signed a petition	26	23	27	1.20 (0.92-1.58)	0.18
Ran for a publicly elected office	0	0	0		0.27
None of these	60	64	59	0.81 (0.64-1.03)	0.09
Do you identify with or actively support any of the following political movements?					
Tea Party (Taxed Enough Already)	6	9	5	0.51 (0.33-0.79)	**<0.01**
Environmental Rights	20	11	23	2.39 (1.66-3.44)	**<0.01**
Women's Rights/ Me Too	23	15	26	2.03 (1.48-2.79)	**<0.01**
Racial Equality	26	17	29	1.99 (1.49-2.66)	**<0.01**
Right to Life	15	23	12	0.46 (0.34-0.62)	**<0.01**
Peace/Anti-War	11	8	12	1.63 (1.08-2.44)	**0.02**
Lesbian, Gay, Bisexual, Transgender, Queer (LGBTQ) Rights	19	10	22	2.60 (1.76-3.84)	**<0.01**
Indivisible	2	2	2	0.94 (0.39-2.24)	0.89
Black Lives Matter	25	16	28	2.04 (1.51-2.75)	**<0.01**
Men's Rights	4	5	4	0.76 (0.44-1.31)	0.32
Alt-right	1	1	0	0.26 (0.07-0.92)	**0.02**
Boogaloo movement	0	0	0	0.43 (0.05-3.47)	0.41
Antifa	2	0	3	48.17 (6.58-352.52)	**<0.01**
QAnon	1	3	0	0.14 (0.05-0.43)	**<0.01**
Anti-gun violence	16	7	19	3.12 (2.11-4.61)	**<0.01**

None of these	53	55	52	0.86 (0.68-1.08)	0.19
Do you identify with or actively support any of the following organizations?					
National Rifle Association (NRA)	13	21	11	0.44 (0.33-0.61)	**<0.01**
Heritage Foundation	3	3	3	1.17 (0.60-2.28)	0.64
Planned Parenthood	19	11	21	2.18 (1.53-3.10)	**<0.01**
National Right to Life Committee	5	8	4	0.56 (0.35-0.90)	**0.01**
Greenpeace	6	4	7	1.72 (0.99-2.97)	0.05
Sierra Club	8	3	9	3.28 (1.69-6.37)	**<0.01**
Amnesty International	6	2	7	4.41 (1.94-10.04)	**<0.01**
National Education Association Foundation	6	6	6	1.00 (0.59-1.68)	0.99
American Civil Liberties Union (ACLU)	12	3	14	4.88 (2.75-8.68)	**<0.01**
Americans for Prosperity	1	3	1	0.25 (0.10-0.61)	**<0.01**
MoveOn.org	7	2	8	3.70 (1.86-7.38)	**<0.01**
The NAACP/National Association for the Advancement of Colored People	12	8	13	1.67 (1.15-2.42)	**0.01**
American Red Cross	23	18	24	1.44 (1.07-1.93)	**0.02**
Chamber of Commerce	3	3	3	1.27 (0.64-2.51)	0.49
Freedom Caucus	2	2	2	0.71 (0.33-1.51)	0.37
None of these	53	57	52	0.82 (0.65-1.04)	0.10
Sources of Health Information					
Which of the following sources have you used to look for health and wellness related information or education in the past 12 months?					
Doctor	60	42	66	2.66 (2.10-3.37)	**<0.01**

Pharmacist	21	14	24	1.87 (1.37-2.57)	**<0.01**
Nurse, nurse practitioner or physician's assistant	29	21	31	1.66 (1.26-2.20)	**<0.01**
Relative, friend or co-worker	23	24	23	0.94 (0.71-1.24)	0.65
Someone you know who has a particular medical condition	8	10	8	0.78 (0.52-1.17)	0.23
Disease-related association or society	5	3	6	2.04 (1.06-3.93)	**0.03**
Patient support group or foundation	2	2	2	1.16 (0.50-2.70)	0.74
Educational forum at a local clinic, hospital, community center or other location	3	4	3	0.83 (0.44-1.57)	0.57
Pharmaceutical company	1	1	1	1.05 (0.38-2.89)	0.92
Health insurance company	7	4	8	2.16 (1.25-3.72)	**<0.01**
Newspapers or magazines	7	4	7	2.07 (1.14-3.77)	**0.02**
Television	6	7	6	0.96 (0.61-1.54)	0.88
The internet	48	43	50	1.30 (1.03-1.65)	**0.03**
Social Media (such as Facebook, Twitter)	6	7	5	0.76 (0.47-1.24)	0.27
Healthcare app for smartphone or tablet	6	6	6	1.04 (0.65-1.67)	0.86
Have not looked for information in the past 12 months	21	33	17	0.40 (0.31-0.52)	**<0.01**
Barriers, Specific Concerns and Other Reasons For Not Getting the Flu Vaccine					
Of those who did not get a flu shot this past year: this is because… [i]					
The flu is not a serious illness	9	12	8	0.60 (0.35-1.02)	0.06
I'm healthy	22	26	20	0.72 (0.50-1.03)	0.07
I just didn't think about it	22	19	25	1.43 (0.98-2.09)	0.07

I didn't know where to get it	1	1	1	1.15 (0.27-4.93)	0.85
I didn't have health insurance	3	3	4	1.47 (0.67-3.21)	0.33
I didn't have time	5	2	8	5.20 (2.17-12.46)	**<0.01**
I don't believe in vaccines	6	12	2	0.11 (0.05-0.22)	**<0.01**
I'm afraid of the side effects	11	15	8	0.50 (0.31-0.80)	**<0.01**
I'm afraid of needles	4	4	5	1.27 (0.61-2.64)	0.52
I prefer alternative (homeopathic) medicine to vaccines	11	15	7	0.43 (0.26-0.71)	**<0.01**
I have never had the flu	13	13	13	0.93 (0.60-1.43)	0.73
The vaccine will make me sick with the flu	9	12	7	0.55 (0.34-0.89)	**0.01**
I got a flu shot the year before so I didn't need it	2	4	1	0.24 (0.10-0.55)	**<0.01**
Another reason	27	27	26	0.95 (0.68-1.33)	0.76

Red text indicates survey items reflecting negative vaccine attitudes

[a] Column percentages (of total sample), weighted according to survey weights to achieve national representativeness

[b] Column percentages (of vaccinated/unvaccinated) (except for first row "All" which is a row percentage), weighted according to survey weights to achieve national representativeness

[c] using the Pearson chi-square test at significance level of alpha=5%; bold indicates statistical significance ($p<0.05$). For non-dichotomous categorical variables, p-values for differences between all categories included in top row with variable name, and p-values for differences between individual categories and reference category included in individual category row.

[e] Likert scale response options (strongly agree, agree, disagree, strongly disagree, don't know) dichotomized to agree/disagree (don't know coded as disagree), results for agreement shown; other scale response options dichotomized to reflect affirmative/negative, results for affirmative shown

[i] asked only to respondents reporting not receiving the flu shot

[j] Odds Ratio (95% Confidence Interval) of being vaccinated vs unvaccinated for affirmative survey response vs not

[k] Reference category for logistic regression of categorical variables
