# Supplementary material for: COVID-19 Vaccination Status, Attitudes, and Values among US Adults in September 2021
Source: J Clin Med. 2022 Jun 28;11(13):3734. doi: 10.3390/jcm11133734 (PMC9267733; doi:10.3390/jcm11133734)
Supplement: Supplementary file 1 [file jcm-11-03734-s001.zip › Table S12.pdf]

Table S12. Frequency and Odds of Intention to Get COVID-19 Vaccine (3 groups) among Unvaccinated by Political Activities and Support, Sources of Health Information, and Reasons For Not Getting the Flu Vaccine

Numbers in the "Total" column indicate the percentage of the unvaccinated weighted sample providing the September 2021 survey response in each row. Numbers in the "Vaccine Intentions" columns indicate the percentage of those whose COVID-19 vaccine intentions match that of the column header who provided the survey response in each row. The numbers in the "OR (95%CI)" columns indicate the Odds Ratio comparing the different vaccine intentions matching that of the column header by the survey response in each row. The numbers in the "P-value" columns indicate the p-value of the associations described in the columns to the left, boldface indicating statistical significance (p<0.05).

	Total Unvacc-inated	Vaccine Intentions, %[b]				Likely vs. Unlikely		Uncertain vs. Unlikely	
**Survey Items**	**(%)[a]**	**Likely**	**Uncertain**	**Unlikely**	**p-value[c]**	**OR (95%CI)[j]**	**p-value[c]**	**OR (95%CI)[j]**	**p-value[c]**
**All**	100	6	55	40					
**Affirmative Responses to Survey Items [e]**									
Political Activities and Support									
People may be involved in civic and political activities. In the past 12 months, have you...									
Attended a political protest	4	4	2	8	**0.01**	0.45 (0.05-3.80)	0.46	0.23 (0.08-0.62)	**<0.01**

or rally									
Contacted a government official	13	23	8	17	**0.03**	1.52 (0.44-5.34)	0.51	0.44 (0.22-0.88)	**0.02**
Volunteered or worked for a Presidential campaign	2	0	2	2	0.83			0.99 (0.18-5.27)	0.99
Volunteered or worked for a political candidate other than a Presidential campaign	1	0	0	3	0.24			0.19 (0.02-1.72)	0.14
Volunteered or worked for a political party, issue, or cause	2	7	1	2	0.20	3.07 (0.33-28.86)	0.32	0.51 (0.12-2.13)	0.36
Served on a committee for a civic, non-profit or community organization	3	1	3	4	0.55	0.21 (0.02-1.90)	0.16	0.78 (0.24-2.61)	0.69
Written a letter or email to a newspaper/magazine or called a live radio or TV show	5	0	4	6	0.54			0.77 (0.26-2.24)	0.62
Commented about politics on a message board or internet site	18	28	14	22	0.11	1.41 (0.46-4.32)	0.54	0.59 (0.34-1.04)	0.07

Shared your opinion about a town or community issue at a public meeting	4	7	2	4	0.45	1.78 (0.20-15.67)	0.60	0.51 (0.12-2.09)	0.35
Held a publicly elected office	0	0	0	0	0.68				
Signed a petition	23	35	20	25	0.25	1.56 (0.57-4.30)	0.38	0.75 (0.45-1.25)	0.27
Ran for a publicly elected office	0	0	0	0					
None of these	64	63	66	63	0.83	1 (0.38-2.60)	0.99	1.15 (0.73-1.80)	0.55
Do you identify with or actively support any of the following political movements?									
Tea Party (Taxed Enough Already)	9	8	7	13	0.18	0.6 (0.09-3.87)	0.59	0.47 (0.22-1.02)	0.06
Environmental Rights	11	19	12	8	0.30	2.66 (0.67-10.5)	0.16	1.55 (0.69-3.45)	0.28
Women's Rights/ Me Too	15	23	16	11	0.24	2.35 (0.75-7.32)	0.14	1.53 (0.79-3.00)	0.21
Racial Equality	17	44	17	12	**<0.01**	5.52 (2.04-14.89)	**<0.01**	1.48 (0.79-2.78)	0.22
Right to Life	23	16	22	26	0.52	0.56 (0.17-1.88)	0.35	0.80 (0.47-1.35)	0.40
Peace/Anti-War	8	15	6	9	0.26	1.73 (0.53-5.71)	0.36	0.67 (0.30-1.51)	0.33
Lesbian, Gay, Bisexual,	10	29	9	8	**0.02**	4.83 (1.44-16.28)	**0.01**	1.15 (0.48-2.78)	0.75

Transgender, Queer									
(LGBTQ) Rights									
Indivisible	2	9	1	2	0.10	4.62 (0.54-39.61)	0.16	0.61 (0.11-3.28)	0.56
Black Lives Matter	16	25	16	13	0.35	2.19 (0.74-6.52)	0.16	1.27 (0.68-2.36)	0.45
Men's Rights	5	4	4	6	0.45	0.56 (0.06-4.85)	0.60	0.56 (0.21-1.50)	0.25
Alt-right	1	0	2	1	0.83			1.38 (0.17-11.08)	0.76
Boogaloo movement	0	0	0	1	0.35			0.11 (0.01-1.80)	0.12
Antifa	0	0	0	0	0.66				
QAnon	3	0	0	7	**<0.01**			0.02 (0.00-0.12)	**<0.01**
Anti-gun violence	7	10	8	5	0.51	1.86 (0.51-6.84)	0.35	1.48 (0.62-3.55)	0.38
None of these	55	40	57	56	0.30	0.52 (0.21-1.26)	0.15	1.04 (0.67-1.62)	0.85
Do you identify with or actively support any of the following organizations?									
National Rifle Association									
(NRA)	21	6	16	31	**<0.01**	0.15 (0.04-0.56)	**<0.01**	0.43 (0.25-0.74)	**<0.01**
Heritage Foundation	3	1	3	3	0.68	0.31 (0.03-2.83)	0.30	0.83 (0.25-2.83)	0.77
Planned Parenthood	11	37	12	6	**<0.01**	10.05 (3.20-31.54)	**<0.01**	2.28 (1.00-5.22)	0.05
National Right to Life									
Committee	8	1	7	9	0.21	0.13 (0.02-1.1)	0.06	0.69 (0.31-1.53)	0.36

Greenpeace	4	12	5	2	0.11	6.86 (0.91-51.97)	0.06	2.52 (0.60-10.60)	0.21
Sierra Club	3	8	3	3	0.38	3.45 (0.40-30.04)	0.26	1.01 (0.26-3.89)	0.99
Amnesty International	2	12	2	0	**<0.01**				
National Education Association Foundation	6	16	7	4	0.16	4.64 (0.87-24.91)	0.07	1.98 (0.59-6.57)	0.27
American Civil Liberties Union (ACLU)	3	13	4	1	**0.01**	18.43 (2.09-162.43)	**0.01**	5.62 (0.98-32.23)	0.05
Americans for Prosperity	3	15	2	2	**0.01**	10.23 (1.33-78.63)	**0.03**	1.44 (0.27-7.61)	0.67
MoveOn.org	2	17	1	1	**<0.01**	16.13 (2.81-92.69)	**<0.01**	1.11 (0.25-4.99)	0.89
The NAACP/National Association for the Advancement of Colored People	8	19	9	5	0.06	4.13 (1.16-14.71)	**0.03**	1.80 (0.80-4.06)	0.16
American Red Cross	18	38	19	13	**0.02**	4.09 (1.48-11.3)	**0.01**	1.59 (0.88-2.88)	0.12
Chamber of Commerce	3	6	4	1	0.11	10.61 (0.63-179.69)	0.10	7.02 (0.86-56.93)	0.07
Freedom Caucus	2	9	2	3	0.12	3.56 (0.53-23.84)	0.19	0.60 (0.15-2.45)	0.48
None of these	57	46	60	54	0.27	0.74 (0.30-1.78)	0.50	1.31 (0.84-2.03)	0.23
Sources of Health Information									
Which of the following									

sources have you used to look for health and wellness related information or education in the past 12 months?									
Doctor	42	42	44	38	0.47	1.17 (0.47-2.89)	0.74	1.31 (0.84-2.04)	0.23
Pharmacist	14	17	17	10	0.13	1.82 (0.51-6.43)	0.35	1.92 (1.02-3.60)	**0.04**
Nurse, nurse practitioner or physician's assistant	21	34	22	18	0.20	2.41 (0.88-6.63)	0.09	1.32 (0.76-2.28)	0.32
Relative, friend or co-worker	24	41	25	20	0.10	2.82 (1.06-7.46)	**0.04**	1.37 (0.80-2.34)	0.26
Someone you know who has a particular medical condition	10	10	13	7	0.28	1.46 (0.23-9.31)	0.69	1.99 (0.88-4.50)	0.10
Disease-related association or society	3	0	2	4	0.46			0.55 (0.15-2.00)	0.36
Patient support group or foundation	2	0	2	2	0.79			1.15 (0.24-5.48)	0.86
Educational forum at a local clinic, hospital, community center or other location	4	7	4	2	0.57	3.12 (0.30-32.27)	0.34	1.66 (0.42-6.59)	0.47
Pharmaceutical company	1	1	2	1	0.56	1.41 (0.08-23.81)	0.81	2.32 (0.24-22.15)	0.46

Health insurance company	4	2	5	2	0.09	0.91 (0.10-.10-8.26)	0.93	2.72 (0.92-8.07)	0.07
Newspapers or magazines	4	2	5	2	0.20	1.06 (0.10-.10-11.59)	0.96	2.76 (0.65-11.78)	0.17
Television	7	9	8	4	0.34	2.32 (0.52-10.4)	0.27	1.92 (0.64-5.78)	0.25
The internet	43	54	44	40	0.48	1.71 (0.71-4.15)	0.23	1.14 (0.74-1.78)	0.55
Social Media (such as Facebook, Twitter)	7	15	6	7	0.37	2.39 (0.56-10.28)	0.24	0.90 (0.35-2.32)	0.82
Healthcare app for smartphone or tablet	6	9	4	8	0.11	1.18 (0.29-4.76)	0.81	0.44 (0.18-1.06)	0.07
Have not looked for information in the past 12 months	33	14	32	38	0.05	0.27 (0.09-0.78)	**0.02**	0.75 (0.48-1.19)	0.22
Barriers, Specific Concerns and Other Reasons For Not Getting the Flu Vaccine									
Of those who did not get a flu shot this past year: this is because… [i]									
The flu is not a serious illness	12	0	10	17	0.08			0.54 (0.26-1.12)	0.10

I'm healthy	26	21	25	27	0.88	0.72 (0.17-3.04)	0.66	0.92 (0.54-1.57)	0.76
I just didn't think about it	19	51	21	13	**<0.01**	6.82 (2.12-21.94)	**<0.01**	1.68 (0.86-3.29)	0.13
I didn't know where to get it	1	0	1	1	0.87			1.57 (0.14-18.17)	0.72
I didn't have health insurance	3	0	4	1	0.13			5.41 (0.67-43.60)	0.11
I didn't have time	2	4	3	0	0.06				
I don't believe in vaccines	12	0	6	22	**<0.01**			0.22 (0.11-0.48)	**<0.01**
I'm afraid of the side effects	15	6	13	18	0.20	0.28 (0.07-1.1)	0.07	0.71 (0.38-1.33)	0.28
I'm afraid of needles	4	3	4	4	0.89	0.84 (0.09-7.95)	0.88	1.21 (0.36-4.07)	0.76
I prefer alternative (homeopathic) medicine to vaccines	15	28	10	19	0.06	1.64 (0.40-6.69)	0.49	0.49 (0.26-0.95)	**0.03**
I have never had the flu	13	0	13	16	0.22			0.76 (0.39-1.47)	0.41
The vaccine will make me sick with the flu	12	3	13	13	0.40	0.23 (0.04-1.17)	0.08	1.01 (0.50-2.04)	0.98
I got a flu shot the year before so I didn't need it	4	1	3	6	0.28	0.18 (0.02-1.7)	0.13	0.53 (0.15-1.81)	0.31
Another reason	27	26	24	31	0.38	0.79 (0.25-2.54)	0.69	0.70 (0.41-1.20)	0.19

Red text indicates survey items reflecting negative vaccine attitudes

[a] Column percentages (of unvaccinated), weighted according to survey weights to achieve national representativeness

[b] Column percentages (of corresponding intention categories) (except for first row "All" which is a row percentage), weighted according to survey weights to achieve national representativeness

[c] using the Pearson chi-square test at significance level of alpha=5%; bold indicates statistical significance ($p<0.05$)

[e] Likert scale response options (strongly agree, agree, disagree, strongly disagree, don't know) dichotomized to agree/disagree (don't know coded as disagree), results for agreement shown; other scale response options dichotomized to reflect affirmative/negative, results for affirmative shown

[i] asked only to respondents reporting not receiving the flu shot

[j] Odds Ratio (95% Confidence Interval) of uncertainty vs unlikeliness to receive COVID-19 vaccine for affirmative survey response vs not

[k] Reference category for logistic regression of categorical variables
