# Supplementary material for: COVID-19 Vaccination Status, Attitudes, and Values among US Adults in September 2021
Source: J Clin Med. 2022 Jun 28;11(13):3734. doi: 10.3390/jcm11133734 (PMC9267733; doi:10.3390/jcm11133734)
Supplement: Supplementary file 1 [file jcm-11-03734-s001.zip › Table S13.pdf]

Table S13. Race/Ethnicity by Political Activities and Support, Sources of Health Information, and Reasons For Not Getting the Flu Vaccine

Numbers in the "Total" column indicate the percentage of the total weighted sample providing the September 2021 survey response in each row. Numbers in the "Race/Ethnicity" columns indicate the percentage of those whose race/ethnicity match that of the column header who provided the survey response in each row. The numbers in the final column indicate the p-value of this association, boldface indicating statistical significance (p<0.05).

		Race/Ethnicity (%)[b]				
**Survey Items**	**Total (%)[a]**	**White, non-Hispanic**	**Black, non-Hispanic**	**Hispanic**	**Other, non-Hispanic**	**p-value[c]**
**All**	100	63	12	17	8	
**Affirmative Responses to Survey Items [e]**						
Political Activities and Support						
People may be involved in civic and political activities. In the past 12 months, have you...						
Attended a political protest or rally	7	7	7	4	10	0.08
Contacted a government official	15	19	8	10	13	**<0.01**
Volunteered or worked for a Presidential campaign	3	3	2	3	3	0.93
Volunteered or worked for a political candidate other than a Presidential campaign	2	3	3	1	3	0.64
Volunteered or worked for a political party, issue, or cause	4	4	4	3	5	0.53
Served on a committee for a civic, non-	5	6	6	2	5	0.15

profit or community organization						
Written a letter or email to a newspaper/magazine or called a live radio or TV show	4	5	3	2	4	0.27
Commented about politics on a message board or internet site	19	21	14	16	20	**0.02**
Shared your opinion about a town or community issue at a public meeting	4	4	4	5	4	0.98
Held a publicly elected office	<1	<1	<1	<1	<1	0.86
Signed a petition	26	26	24	24	29	0.57
Ran for a publicly elected office	<1	<1	<1	<1	<1	0.59
None of these	60	59	62	67	59	**0.04**
Do you identify with or actively support any of the following political movements?						
Tea Party (Taxed Enough Already)	6	8	3	3	6	**<0.01**
Environmental Rights	20	19	20	21	24	0.51
Women's Rights/ Me Too	23	21	33	21	32	**<0.01**
Racial Equality	26	23	46	23	32	**<0.01**
Right to Life	15	16	13	12	12	0.21
Peace/Anti-War	11	11	12	12	12	0.86
Lesbian, Gay, Bisexual, Transgender, Queer (LGBTQ) Rights	19	19	19	19	23	0.69
Indivisible	2	2	2	1	3	0.37
Black Lives Matter	25	20	52	22	33	**<0.01**
Men's Rights	4	3	8	5	3	**0.03**
Alt-right	1	1	1	<1	1	0.72
Boogaloo movement	<1	<1	0	<1	0	0.68
Antifa	2	2	2	3	1	0.60

QAnon	1	1	<1	<1	1	**0.03**
Anti-gun violence	16	14	23	17	20	**0.01**
None of these	53	54	38	60	46	**<0.01**
Do you identify with or actively support any of the following organizations?						
National Rifle Association (NRA)	13	17	3	7	9	**<0.01**
Heritage Foundation	3	4	4	3	2	0.79
Planned Parenthood	19	18	21	16	26	0.08
National Right to Life Committee	5	6	2	4	4	**0.03**
Greenpeace	6	6	8	6	6	0.88
Sierra Club	8	8	4	6	10	0.11
Amnesty International	6	6	6	6	7	0.91
National Education Association Foundation	6	7	8	4	6	0.30
American Civil Liberties Union (ACLU)	12	12	13	10	13	0.62
Americans for Prosperity	1	1	2	1	1	0.58
MoveOn.org	7	7	6	5	8	0.52
The NAACP/National Association for the Advancement of Colored People	12	8	40	10	10	**<0.01**
American Red Cross	23	23	26	18	25	0.21
Chamber of Commerce	3	3	4	3	3	0.84
Freedom Caucus	2	2	4	2	1	0.08
None of these	53	51	49	63	52	**<0.01**

Sources of Health Information

Which of the following sources have you used to look for health and wellness related information or education in the

past 12 months?						
Doctor	60	64	55	49	60	**<0.01**
Pharmacist	21	23	19	17	20	0.13
Nurse, nurse practitioner or physician's assistant	29	33	24	18	19	**<0.01**
Relative, friend or co-worker	23	24	21	19	29	0.11
Someone you know who has a particular medical condition	8	9	8	8	7	0.71
Disease-related association or society	5	6	5	5	7	0.84
Patient support group or foundation	2	2	2	2	1	0.87
Educational forum at a local clinic, hospital, community center or other location	3	2	6	5	5	**0.01**
Pharmaceutical company	1	1	3	<1	0	**0.03**
Health insurance company	7	6	9	7	7	0.43
Newspapers or magazines	7	6	8	6	8	0.40
Television	6	5	11	10	3	**<0.01**
The internet	48	49	41	47	53	0.10
Social Media (such as Facebook, Twitter)	6	5	4	10	5	**0.01**
Healthcare app for smartphone or tablet	6	5	7	7	7	0.38
Have not looked for information in the past 12 months	21	20	22	22	17	0.60

Barriers, Specific Concerns and Other Reasons For Not Getting the Flu Vaccine

Of those who did not get a flu shot this past year: this is because… [i]

The flu is not a serious illness	9	13	2	4	11	**<0.01**
I'm healthy	22	24	14	22	30	0.07
I just didn't think about it	22	22	21	22	30	0.52
I didn't know where to get it	1	1	1	3	0	0.11
I didn't have health insurance	3	2	5	6	1	**0.02**
I didn't have time	5	4	2	9	9	**0.04**
I don't believe in vaccines	6	7	8	4	1	0.06
I'm afraid of the side effects	11	10	14	9	19	0.09
I'm afraid of needles	4	5	6	4	1	0.40
I prefer alternative (homeopathic) medicine to vaccines	11	12	8	10	11	0.63
I have never had the flu	13	15	12	10	11	0.45
The vaccine will make me sick with the flu	9	9	13	8	9	0.46
I got a flu shot the year before so I didn't need it	2	2	5	3	2	0.15
Another reason	27	29	31	21	19	0.11

Red text indicates survey items reflecting negative vaccine attitudes

[a] Column percentages (of total sample), weighted according to survey weights to achieve national representativeness

[b] Column percentages (of race/ethnicity) (except for first row "All" which is a row percentage), weighted according to survey weights to achieve national representativeness

[c] using the Pearson chi-square test at significance level of alpha=5%; bold indicates statistical significance ($p<0.05$)

[e] Likert scale response options (strongly agree, agree, disagree, strongly disagree, don't know) dichotomized to agree/disagree (don't know coded as disagree), results for agreement shown; other scale response options dichotomized to reflect affirmative/negative, results for affirmative shown

[i] asked only to respondents reporting not receiving the flu shot
