# Supplementary material for: COVID-19 Vaccination Status, Attitudes, and Values among US Adults in September 2021
Source: J Clin Med. 2022 Jun 28;11(13):3734. doi: 10.3390/jcm11133734 (PMC9267733; doi:10.3390/jcm11133734)
Supplement: Supplementary file 1 [file jcm-11-03734-s001.zip › Table S14.pdf]

Table S14. Race/Ethnicity among Unvaccinated and Uncertain by Vaccine Attitudes and Trust in CDC and HDs

Numbers in the "Total" column indicate the percentage of the total weighted sample who were unvaccinated and uncertain providing the September 2021 survey response in each row. Numbers in the "Race/Ethnicity" columns indicate the percentage of those unvaccinated and uncertain whose race/ethnicity match that of the column header who provided the survey response in each row. The numbers in the final column indicate the p-value of this association, boldface indicating statistical significance (p<0.05).

		Race/Ethnicity (%)[b]				
**Survey Items**	**Total (%)[a]**	**White, non-Hispanic**	**Black, non-Hispanic**	**Hispanic**	**Other, non-Hispanic**	**p-value[c]**
**All**	100	63	16	17	4	
**Constructs [d]**						
Confidence in vaccines	18	18	20	17	0	0.52
Trust in the Centers for Disease Control and Prevention (CDC)	14	13	20	16	0	0.30
Trust in local and state health departments (HDs)	19	19	28	15	0	0.19
**Affirmative Responses to Survey Items [e]**						
COVID-19 Disease						
Have you ever had COVID-19?	27	25	29	27	39	0.86
How likely do you think it is that you will have COVID-19 over the next year?	27	25	28	28	47	0.53
When indoors in a crowded setting do you (or would you) wear a mask?	70	62	91	81	72	**<0.01**
I am concerned that I or my	46	35	77	56	65	**<0.01**

family/friends will be exposed when others do not wear masks in public.						
COVID-19 Vaccine						
How important do you think a COVID-19 vaccine is to stop the spread of infection in the US?	50	48	66	52	21	**0.03**
Are you worried that the COVID-19 vaccine is not safe for adults?	68	70	64	59	90	0.12
Have you discussed getting vaccinated with your healthcare provider?	29	34	19	17	47	**0.04**
Of those who have: the provider encouraged getting the vaccine.	30	32	33	30	9	0.62
COVID-19 in Children						
COVID-19 can be a serious disease for some children.	74	70	84	78	90	0.08
I am concerned about the safety of COVID-19 vaccine in children.	87	88	87	83	92	0.77
Vaccinating children against COVID-19 is important to end the pandemic and get back to normal.	26	21	44	33	0	**0.01**
It is better for children to develop immunity to COVID-19 by getting sick rather than by getting a shot.	61	67	42	57	72	0.05
COVID-19 in children is no worse than a cold or the flu.	53	56	38	55	45	0.21

Vaccines Other than COVID-19						
Had flu vaccination, past 12 months.	21	23	17	21	13	0.75
Of parents: Have you ever delayed having your child get a shot other than the flu for reasons other than illness or allergy?	28	35	15	10	58	0.08
Of parents: Have you ever decided not to have your child get a shot other than the flu for reasons other than illness or allergy?	23	20	32	6	70	**0.02**
Have you or anyone you know ever had a serious reaction to a vaccine?	21	24	11	16	33	0.25
Healthcare and Science in General						
Received high quality care from healthcare provider, past 12 months.	85	90	73	72	86	**0.01**
In general, would you say that you trust science?	77	78	76	77	79	0.99
Of Unvaccinated: Mandates and Incentives [i]						
Of employed: If my employer required me to get the COVID-19 vaccine... I would get vaccinated. [i]	16	17	20	17	0	0.57
Of those who would not:						
I would quit my job. [i]	16	15	22	13	16	0.86
I would protest. [i]	22	22	12	32	11	0.17
I would consider legal action. [i]	29	31	17	27	40	0.50
I am not sure what I would do. [i]	57	57	57	59	60	0.99
If I was offered a $25-$100 gift card for	7	8	2	5	8	0.42

getting fully vaccinated... I would be more likely to get vaccinated. [i]						
If I was automatically enrolled in a lottery when I got fully vaccinated that made me eligible to win at least $100K... I would be more likely to get vaccinated. [i]	7	5	11	8	0	0.47
Seeing fewer people wear masks in public makes me more likely to get vaccinated. [i]	15	11	27	18	25	0.14
Of Unvaccinated: Knowledge and Decision-Making re: COVID-19 Vaccination [i]						
I am knowledgeable about COVID-19 vaccines for adults. [i]	72	70	79	68	96	0.09
I still have many unanswered questions about COVID-19 vaccines for adults. [i]	76	76	74	74	96	0.34
I still cannot decide whether getting the COVID-19 vaccine is best for me. [i]	70	69	68	73	76	0.89
Talking with other people is important in helping me make up my mind about COVID-19 vaccination for myself. [i]	43	40	50	41	78	0.05
Of Unvaccinated: Specific Concerns and Other Reasons For Not Getting a COVID-19 Vaccine [i]						
How fast COVID-19 vaccines were developed and made available to the public. [i]	57	59	43	60	71	0.27
COVID-19 vaccines are new. [i]	79	82	70	74	81	0.24

The safety of COVID-19 vaccines has not been studied for a long enough period of time. [i]	81	85	69	75	96	**0.03**
A lot of people who get the vaccine feel tired, achy and get headaches and fever. [i]	74	76	62	77	78	0.29
Some people have bad allergic reactions to COVID-19 vaccines. [i]	74	76	69	68	96	0.13
I am not sure the ingredients in COVID-19 vaccines are safe. [i]	73	75	65	69	96	0.12
COVID-19 vaccines might change my genes or DNA (cause mutations). [i]	24	23	26	19	43	0.38
COVID-19 vaccines might affect my fertility or ability to have children. [i]	26	23	30	35	37	0.37
There were not enough people of my race/ethnicity who were a part of the vaccine studies. [i]	17	11	28	25	42	**0.01**
They are experimenting on people with the COVID-19 vaccine. [i]	61	59	48	70	96	**<0.01**
The drug companies are making a lot of money off of COVID-19 vaccines. [i]	69	70	63	64	88	0.31
Some COVID-19 vaccines are made from aborted fetuses. [i]	17	17	18	14	35	0.51
I have a health condition that might make me at increased risk of having a bad reaction to the COVID-19 vaccine. [i]	32	32	31	24	69	**0.03**
I have a health condition that would prevent the COVID-19 vaccine from being effective. [i]	18	17	12	18	44	0.12

I am worried about severe vaccine side effects such as myocarditis (heart swelling), Guillain Barre Syndrome (paralysis), or severe blood clots. [i]	73	69	76	83	83	0.19
Vaccine recommendations are influenced more by politics than by science. [i]	79	83	69	73	92	0.08
I am worried about the safety of COVID-19 vaccines. [i]	86	86	83	83	100	0.48
I do not trust how quickly the COVID-19 vaccine was developed. [i]	86	88	74	86	96	0.05
I worry that I would have a reaction to the vaccine. [i]	81	79	82	84	91	0.59
I worry about having to provide personal information (name, address, phone number, insurance card) to get the vaccine. [i]	23	19	24	33	32	0.32
Those I trust (friends, family, or religious leaders) do not want to get the vaccine. [i]	47	51	40	37	63	0.21
I have seen posts on social media that make me wary of the vaccine. [i]	54	50	57	58	74	0.31
I want to wait to see what happens to others who are vaccinated. [i]	81	80	83	81	87	0.87
I need more time to learn and think more about it. [i]	80	79	84	79	87	0.83
Of pregnant women: I do not think the COVID-19 vaccine is safe for me or my baby. [i]	41	61	26	4	41	0.05

Of Unvaccinated: Reasons to Get COVID-19 Vaccination [i]						
COVID-19 vaccines are likely to protect me from the COVID-19 strains circulating. [i]	34	34	38	35	8	0.24
COVID-19 vaccines are likely to protect me from new variants of COVID-19 that may appear in the future. [i]	29	25	40	38	8	0.05
It's important for me to get vaccinated so I don't accidentally give COVID-19 to other people in my family. [i]	29	24	47	33	34	0.07
It's important for me to get vaccinated to help get my community back to normal. [i]	20	16	37	25	0	**0.01**
The government is acting in my or my family's best interest when it comes to COVID-19. [i]	23	19	32	35	8	**0.04**
Of Unvaccinated: Barriers to COVID-19 Vaccination [i]						
I cannot get transportation to where COVID-19 vaccines are being given. [i]	7	7	6	8	0	0.80
The times when COVID-19 vaccines are being given conflict with my daily schedule. [i]	12	9	26	16	0	**0.03**
I do not know how to register to get a vaccine appointment. [i]	11	11	13	13	0	0.67
I know how to register to get a vaccine appointment but it is too difficult. [i]	18	13	33	17	29	0.05

I cannot miss work to get vaccinated. [i]	19	16	24	20	37	0.31
At least one of the above. [i]	34	28	49	35	65	**0.01**

Red text indicates survey items reflecting negative vaccine attitudes

[a] Column percentages (of total sample), weighted according to survey weights to achieve national representativeness

[b] Column percentages (of race/ethnicity) (except for first row "All" which is a row percentage), weighted according to survey weights to achieve national representativeness

[c] using the Pearson chi-square test at significance level of alpha=5%; bold indicates statistical significance (p<0.05)

[d] Construct scales combine scores for each relevant survey item (reversing negative items) and divide by maximum (e.g., 100 being complete trust and 0 being complete distrust); after dichotomizing at median, binary variable represents high vs low score (e.g., 1 being high trust and 0 being low trust)

[e] Likert scale response options (strongly agree, agree, disagree, strongly disagree, don't know) dichotomized to agree/disagree (don't know coded as disagree), results for agreement shown; other scale response options dichotomized to reflect affirmative/negative, results for affirmative shown

[i] asked only to unvaccinated respondents

[j] asked only to vaccinated respondents
