# Supplementary material for: COVID-19 Vaccination Status, Attitudes, and Values among US Adults in September 2021
Source: J Clin Med. 2022 Jun 28;11(13):3734. doi: 10.3390/jcm11133734 (PMC9267733; doi:10.3390/jcm11133734)
Supplement: Supplementary file 1 [file jcm-11-03734-s001.zip › Table S2.pdf]

Table S2. Frequency and Odds of Hesitation Before Getting COVID-19 Vaccine among Vaccinated by Vaccine Attitudes, Trust in CDC and HDs, and Sociodemographic Characteristics

Numbers in the "Total" column indicate the percentage of the vaccinated weighted sample providing the September 2021 survey response in each row. Numbers in the "Hesitation Prior to Vaccination" columns indicate the percentage of those whose amount of hesitation prior to receiving the COVID-19 vaccine match that of the column header who provided the survey response in each row. The numbers in the "OR (95%CI)" columns indicate the Odds Ratio comparing the different amounts of hesitation matching that of the column header by the survey response in each row. The numbers in the "P-value" columns indicate the p-value of the associations described in the columns to the left, boldface indicating statistical significance (p<0.05).

Survey Items	Total Vaccinated (%)[a]	Hesitation Prior to Vaccination, %[b]				No Hesitation vs. Some Hesitation		No Hesitation vs. A Lot of Hesitation	
		None	Some	A Lot	p-value[c]	OR (95%CI)[j]	p-value[c]	OR (95%CI)[j]	p-value[c]
**All**	100	65	24	11					
**Constructs** [d]									
Confidence in vaccines	79	94	65	27	**<0.01**	8.70 (5.97-12.67)	**<0.01**	43.09 (26.97-68.86)	**<0.01**
Trust in the Centers for Disease Control and Prevention (CDC)	58	70	42	23	**<0.01**	3.20 (2.45-4.18)	**<0.01**	7.53 (5.05-11.22)	**<0.01**
Trust in local and state health departments (HDs)	55	64	44	25	**<0.01**	2.28 (1.75-2.96)	**<0.01**	5.28 (3.61-7.72)	**<0.01**
**Sociodemographic Characteristics**									

Gender					**0.01**				
Female	51	48	55	59		ref [k]		ref [k]	
Male	49	52	45	41		1.31 (1.01-1.70)	**0.04**	1.55 (1.09-2.21)	**0.01**
Age (years)					**<0.01**				
18-29	17	16	22	16		ref [k]		ref [k]	
30-44	23	21	27	31		1.03 (0.67-1.60)	0.88	0.65 (0.36-1.20)	0.17
45-59	25	24	26	30		1.26 (0.82-1.93)	0.29	0.76 (0.42-1.37)	0.36
60+	35	40	26	23		2.10 (1.39-3.17)	**<0.01**	1.69 (0.93-3.07)	0.08
Education (attained)					**<0.01**				
<High School	8	7	10	12		ref [k]		ref [k]	
High School	25	22	29	32		1.15 (0.69-1.90)	0.59	1.24 (0.66-2.32)	0.50
Some College	27	27	27	28		1.54 (0.94-2.54)	0.09	1.77 (0.96-3.28)	0.07
Bachelors or Higher	40	45	34	28		2.05 (1.26-3.34)	**<0.01**	2.87 (1.55-5.32)	**<0.01**
Race/Ethnicity					**<0.01**				
White, non-Hispanic	63	67	54	62		ref [k]		ref [k]	
Black, non-Hispanic	11	8	15	17		0.43 (0.31-0.58)	**<0.01**	0.42 (0.27-0.64)	**<0.01**
Hispanic	17	16	20	15		0.65 (0.48-0.87)	**<0.01**	1.00 (0.66-1.51)	0.99

Other, non-Hispanic	10	10	11	6		0.69 (0.40-1.19)	0.18	1.49 (0.62-3.61)	0.37
Region					0.24				
Northeast	18	18	17	24		ref [k]		ref [k]	
Midwest	20	21	18	24		1.13 (0.72-1.75)	0.60	1.16 (0.69-1.94)	0.58
South	37	36	39	34		0.90 (0.62-1.31)	0.59	1.42 (0.88-2.27)	0.15
West	25	25	25	18		0.97 (0.65-1.47)	0.90	1.84 (1.08-3.14)	**0.02**
Metropolitan Statistical Area status (metro vs non-metro)	88	89	88	85	0.42	1.10 (0.71-1.71)	0.66	1.43 (0.84-2.43)	0.19
Employment status (working vs not working)	61	58	65	65	0.05	0.75 (0.57-0.98)	**0.03**	0.75 (0.52-1.08)	0.12
Household income					**<0.01**				
<$50k	26	22	35	31		ref [k]		ref [k]	
$50-85k	31	30	31	39		1.52 (1.10-2.10)	**0.01**	1.08 (0.71-1.64)	0.73
$85-150k	19	20	15	19		2.13 (1.43-3.18)	**<0.01**	1.50 (0.89-2.51)	0.13
$150k+	23	27	18	11		2.38 (1.63-3.47)	**<0.01**	3.55 (1.95-6.45)	**<0.01**
Household size					**0.04**				
1	18	18	16	19		ref [k]		ref [k]	

2	38	40	33	39		1.13 (0.77-1.66)	0.52	1.11 (0.69-1.78)	0.67
3	18	19	18	15		1.00 (0.65-1.54)	0.99	1.44 (0.79-2.62)	0.24
4+	26	23	33	27		0.66 (0.44-0.98)	**0.04**	0.94 (0.56-1.59)	0.83
Number of children (ages 2-17)					**<0.01**				
0	76	79	68	76		ref [k]		ref [k]	
1	10	9	11	12		0.65 (0.43-0.99)	**0.04**	0.68 (0.39-1.20)	0.18
2	10	9	13	8		0.59 (0.39-0.90)	**0.02**	1.05 (0.55-2.00)	0.88
3+	5	4	8	4		0.40 (0.24-0.68)	**<0.01**	0.87 (0.39-1.96)	0.74
Political affiliation					**<0.01**				
Republican	24	20	27	35		ref [k]		ref [k]	
Democrat	39	45	32	22		1.83 (1.31-2.57)	**<0.01**	3.40 (2.13-5.42)	**<0.01**
Independent	29	29	29	26		1.31 (0.91-1.88)	0.15	1.93 (1.22-3.05)	**0.01**
Something else	8	6	11	17		0.75 (0.45-1.25)	0.27	0.63 (0.35-1.12)	0.12
Physical health (good vs not good)	84	86	82	78	**0.04**	1.3 (0.93-1.82)	0.12	1.67 (1.09-2.55)	**0.02**
**Affirmative Responses to Survey Items [e]**									
COVID-19 Disease									

Have you ever had COVID-19?	15	13	17	22	**0.01**	0.72 (0.50-1.05)	0.09	0.53 (0.34-0.84)	**0.01**
How likely do you think it is that you will have COVID-19 over the next year?	18	18	18	22	0.47	0.95 (0.68-1.33)	0.76	0.76 (0.49-1.18)	0.23
When indoors in a crowded setting do you (or would you) wear a mask?	86	88	86	79	**0.03**	1.12 (0.76-1.64)	0.56	1.85 (1.17-2.93)	**0.01**
I am concerned that I or my family/friends will be exposed when others do not wear masks in public.	68	75	61	43	**<0.01**	1.94 (1.46-2.57)	**<0.01**	4.11 (2.85-5.93)	**<0.01**
COVID-19 Vaccine									
How important do you think a COVID-19 vaccine is to stop the spread of infection in the US?	96	99	96	76	**<0.01**	6.21 (2.12-18.18)	**<0.01**	44.28 (16.76-116.99)	**<0.01**
Are you worried that the COVID-19 vaccine is not safe for adults?	11	3	16	50	**<0.01**	0.15 (0.09-0.25)	**<0.01**	0.03 (0.02-0.05)	**<0.01**
Have you discussed getting vaccinated with your healthcare provider?	39	40	34	36	0.14	1.30 (0.99-1.70)	0.06	1.19 (0.83-1.70)	0.35
Of those who have: the provider encouraged getting the vaccine.	79	87	66	52	**<0.01**	3.37 (2.04-5.57)	**<0.01**	5.99 (3.25-11.06)	**<0.01**

COVID-19 in Children									
COVID-19 can be a serious disease for some children.	92	94	89	83	**<0.01**	2.00 (1.25-3.19)	**<0.01**	3.35 (2.02-5.58)	**<0.01**
I am concerned about the safety of COVID-19 vaccine in children.	54	41	73	85	**<0.01**	0.26 (0.19-0.34)	**<0.01**	0.12 (0.08-0.19)	**<0.01**
Vaccinating children against COVID-19 is important to end the pandemic and get back to normal.	85	93	79	52	**<0.01**	3.56 (2.43-5.20)	**<0.01**	12.53 (8.28-18.96)	**<0.01**
It is better for children to develop immunity to COVID-19 by getting sick rather than by getting a shot.	17	10	22	46	**<0.01**	0.42 (0.29-0.59)	**<0.01**	0.14 (0.09-0.20)	**<0.01**
COVID-19 in children is no worse than a cold or the flu.	23	16	30	47	**<0.01**	0.45 (0.33-0.61)	**<0.01**	0.21 (0.14-0.30)	**<0.01**
Vaccines Other than COVID-19									
Had flu vaccination, past 12 months.	66	75	54	38	**<0.01**	2.61 (1.99-3.43)	**<0.01**	4.96 (3.44-7.14)	**<0.01**
Of parents: Have you ever delayed having your child get a shot other than the flu for reasons other than illness or allergy?	9	8	12	9	0.53	0.63 (0.28-1.43)	0.27	0.83 (0.24-2.86)	0.77
Of parents: Have you ever decided not to have	6	3	9	12	**0.02**	0.31 (0.11-0.81)	**0.02**	0.23 (0.07-0.75)	**0.02**

your child get a shot other than the flu for reasons other than illness or allergy?										
Have you or anyone you know ever had a serious reaction to a vaccine?	4	3	5	12	**<0.01**	0.46 (0.25-0.85)	**0.01**	0.19 (0.10-0.37)	**<0.01**
Healthcare and Science in General									
Received high quality care from healthcare provider, past 12 months.	93	95	91	82	**<0.01**	1.83 (1.09-3.07)	**0.02**	4.4 (2.48-7.81)	**<0.01**
In general, would you say that you trust science?	95	98	92	81	**<0.01**	4.02 (2.09-7.75)	**<0.01**	10.88 (5.7-20.77)	**<0.01**
Among Vaccinated: Boosters [j]									
If the CDC were to recommend a booster dose so your body can continue to protect you against COVID-19, how likely are you to get one? [i]	87	97	79	46	**<0.01**	8.1 (5.06-12.98)	**<0.01**	35.86 (21.69-59.31)	**<0.01**
Political Activities and Support									
People may be involved in civic and political activities. In the past 12 months, have you...									
Attended a political protest or rally	7	9	6	3	**0.02**	1.58 (0.90-2.78)	0.11	3.19 (1.33-7.63)	**0.01**
Contacted a government official	16	19	11	11	**<0.01**	1.94 (1.32-2.87)	**<0.01**	1.88 (1.11-3.19)	**0.02**

Volunteered or worked for a Presidential campaign	3	4	1	1	**0.01**	3.8 (1.51-9.57)	**<0.01**	3.43 (0.77-15.33)	0.11
Volunteered or worked for a political candidate other than a Presidential campaign	3	4	1	2	**0.02**	4.15 (1.51-11.42)	**0.01**	1.64 (0.56-4.82)	0.37
Volunteered or worked for a political party, issue, or cause	4	6	2	2	**<0.01**	3.19 (1.43-7.13)	**<0.01**	3.19 (1.03-9.84)	0.04
Served on a committee for a civic, non-profit or community organization	6	7	4	3	**0.01**	1.97 (1.09-3.53)	**0.02**	2.64 (1.13-6.15)	**0.02**
Written a letter or email to a newspaper/magazine or called a live radio or TV show	4	4	4	5	0.88	1.11 (0.57-2.17)	0.76	0.87 (0.38-1.98)	0.74
Commented about politics on a message board or internet site	20	22	16	17	**0.03**	1.52 (1.07-2.16)	**0.02**	1.37 (0.88-2.12)	0.16
Shared your opinion about a town or community issue at a public meeting	4	5	4	3	0.51	1.28 (0.71-2.33)	0.41	1.56 (0.61-3.99)	0.35
Held a publicly elected office	0	1	0	0	0.24	4.61 (0.72-29.60)	0.11		
Signed a petition	27	28	25	25	0.62	1.13 (0.84-1.51)	0.43	1.15 (0.78-1.70)	0.47
Ran for a publicly elected office	0	0	0	0	0.70	1.38 (0.17-11.35)	0.77		
None of these	59	57	64	63	**0.03**	0.73 (0.56-0.95)	**0.02**	0.75 (0.53-1.07)	0.12
Do you identify with or actively support any of									

the following political movements?									
Tea Party (Taxed Enough Already)	5	4	7	9	**<0.01**	0.49 (0.28-0.86)	**0.01**	0.38 (0.19-0.73)	**<0.01**
Environmental Rights	23	27	17	10	**<0.01**	1.76 (1.27-2.44)	**<0.01**	3.16 (1.92-5.21)	**<0.01**
Women's Rights/ Me Too	26	30	21	15	**<0.01**	1.61 (1.18-2.20)	**<0.01**	2.46 (1.53-3.95)	**<0.01**
Racial Equality	29	32	27	17	**<0.01**	1.29 (0.97-1.73)	0.08	2.33 (1.51-3.59)	**<0.01**
Right to Life	12	10	16	17	**<0.01**	0.58 (0.40-0.83)	**<0.01**	0.53 (0.33-0.86)	**0.01**
Peace/Anti-War	12	13	14	8	0.15	0.87 (0.60-1.27)	0.47	1.72 (0.89-3.33)	0.11
Lesbian, Gay, Bisexual, Transgender, Queer (LGBTQ) Rights	22	26	17	13	**<0.01**	1.79 (1.26-2.53)	**<0.01**	2.47 (1.48-4.12)	**<0.01**
Indivisible	2	2	2	0	0.15	1.06 (0.40-2.85)	0.90	15.33 (2.04-115.29)	**0.01**
Black Lives Matter	28	32	23	15	**<0.01**	1.61 (1.19-2.17)	**<0.01**	2.58 (1.67-4.01)	**<0.01**
Men's Rights	4	4	5	4	0.68	0.77 (0.41-1.43)	0.40	0.88 (0.39-1.97)	0.76
Alt-right	0	0	0	1	0.34			0.60 (0.09-3.96)	0.59
Boogaloo movement	0	0	0	1	**0.03**			0.11 (0.01-1.15)	0.07
Antifa	3	3	3	1	0.18	1.30 (0.58-2.89)	0.53	3.93 (0.93-16.69)	0.06
QAnon	0	0	1	1	0.06	0.15 (0.01-1.69)	0.12	0.08 (0.01-0.93)	**0.04**
Anti-gun violence	19	22	15	10	**<0.01**	1.64 (1.16-2.30)	**<0.01**	2.68 (1.54-4.66)	**<0.01**
None of these	52	49	54	62	**0.01**	0.81 (0.63-1.05)	0.12	0.57 (0.40-0.82)	**<0.01**
Do you identify with or actively support any of the following organizations?									

National Rifle Association (NRA)	11	9	13	18	**<0.01**	0.61 (0.41-0.90)	**0.01**	0.43 (0.26-0.69)	**<0.01**
Heritage Foundation	3	3	5	6	0.06	0.54 (0.28-1.02)	0.06	0.44 (0.19-1.02)	0.06
Planned Parenthood	21	25	15	11	**<0.01**	1.95 (1.37-2.78)	**<0.01**	2.75 (1.57-4.81)	**<0.01**
National Right to Life Committee	4	3	6	6	**0.02**	0.49 (0.27-0.87)	**0.02**	0.51 (0.25-1.06)	0.07
Greenpeace	7	8	4	5	**0.03**	2.12 (1.22-3.67)	**0.01**	1.74 (0.76-4.02)	0.19
Sierra Club	9	11	7	2	**<0.01**	1.69 (1.02-2.82)	**0.04**	4.93 (1.82-13.38)	**<0.01**
Amnesty International	7	9	5	2	**<0.01**	1.92 (1.09-3.38)	**0.02**	6.45 (2.03-20.52)	**<0.01**
National Education Association Foundation	6	7	5	4	0.22	1.40 (0.80-2.42)	0.24	2.02 (0.73-5.57)	0.17
American Civil Liberties Union (ACLU)	14	19	7	4	**<0.01**	2.91 (1.86-4.56)	**<0.01**	5.06 (2.57-9.94)	**<0.01**
Americans for Prosperity	1	0	1	2	**0.02**	0.25 (0.06-1.03)	0.05	0.14 (0.03-0.66)	**0.01**
MoveOn.org	8	10	4	2	**<0.01**	2.62 (1.46-4.72)	**<0.01**	4.89 (1.97-12.11)	**<0.01**
The NAACP/National Association for the Advancement of Colored People	13	15	11	9	**0.02**	1.44 (1.00-2.07)	0.05	1.80 (1.05-3.09)	**0.03**
American Red Cross	24	26	22	17	**0.03**	1.26 (0.93-1.71)	0.14	1.73 (1.09-2.74)	**0.02**
Chamber of Commerce	3	4	3	3	0.65	1.30 (0.65-2.60)	0.45	1.41 (0.50-4.00)	0.52
Freedom Caucus	2	1	3	2	0.27	0.50 (0.22-1.16)	0.11	0.83 (0.22-3.17)	0.79
None of these	52	49	54	60	**0.03**	0.83 (0.64-1.07)	0.15	0.64 (0.45-0.91)	**0.01**

Sources of Health Information

Which of the following sources have you used to

look for health and wellness related information or education in the past 12 months?										
Doctor	66	69	60	56	**<0.01**	1.49 (1.14-1.95)	**<0.01**	1.77 (1.24-2.52)	**<0.01**
Pharmacist	24	25	21	21	0.22	1.29 (0.94-1.77)	0.12	1.25 (0.82-1.92)	0.30
Nurse, nurse practitioner or physician's assistant	31	33	27	28	0.11	1.32 (0.99-1.77)	0.06	1.26 (0.86-1.86)	0.23
Relative, friend or co-worker	23	23	24	25	0.79	0.93 (0.68-1.27)	0.64	0.88 (0.59-1.33)	0.55
Someone you know who has a particular medical condition	8	7	9	10	0.35	0.75 (0.47-1.19)	0.22	0.74 (0.40-1.34)	0.31
Disease-related association or society	6	7	7	3	0.19	0.94 (0.57-1.55)	0.81	2.08 (0.98-4.39)	0.06
Patient support group or foundation	2	2	2	1	0.49	0.95 (0.42-2.15)	0.91	2.47 (0.55-11.14)	0.24
Educational forum at a local clinic, hospital, community center or other location	3	3	3	5	0.27	0.95 (0.48-1.91)	0.89	0.55 (0.26-1.17)	0.12
Pharmaceutical company	1	1	1	2	0.89	1.22 (0.44-3.41)	0.70	0.89 (0.27-2.98)	0.85
Health insurance company	8	8	9	5	0.31	0.92 (0.57-1.46)	0.71	1.64 (0.87-3.08)	0.13
Newspapers or magazines	7	8	6	6	0.40	1.38 (0.83-2.32)	0.22	1.28 (0.66-2.49)	0.47
Television	6	7	6	6	0.69	1.2 (0.73-1.99)	0.47	1.22 (0.63-2.33)	0.56
The internet	50	51	48	45	0.26	1.14 (0.88-1.47)	0.33	1.3 (0.92-1.84)	0.14
Social Media (such as Facebook, Twitter)	5	4	7	7	0.07	0.57 (0.33-0.97)	**0.04**	0.59 (0.30-1.16)	0.13
Healthcare app for smartphone or tablet	6	6	5	9	0.23	1.26 (0.69-2.29)	0.45	0.65 (0.35-1.22)	0.18

Have not looked for information in the past 12 months	17	14	20	24	**<0.01**	0.65 (0.46-0.91)	**0.01**	0.52 (0.34-0.81)	**<0.01**
Barriers, Specific Concerns and Other Reasons For Not Getting the Flu Vaccine									
Of those who did not get a flu shot this past year: this is because… [i]									
The flu is not a serious illness	8	6	8	11	0.37	0.71 (0.28-1.80)	0.47	0.50 (0.20-1.28)	0.15
I'm healthy	20	17	24	22	0.29	0.65 (0.37-1.15)	0.14	0.71 (0.37-1.35)	0.29
I just didn't think about it	25	29	28	11	**<0.01**	1.07 (0.65-1.74)	0.79	3.43 (1.59-7.42)	**<0.01**
I didn't know where to get it	1	2	0	0	0.07	6.57 (1.01-42.58)	**0.05**		
I didn't have health insurance	4	5	4	0	**0.02**	1.47 (0.60-3.60)	0.40	17.58 (2.20-140.51)	**0.01**
I didn't have time	8	10	10	1	**0.02**	1.06 (0.50-2.27)	0.88	8.28 (1.85-37.03)	**0.01**
I don't believe in vaccines	2	0	1	5	**<0.01**	0.27 (0.05-1.43)	0.12	0.06 (0.01-0.28)	**<0.01**
I'm afraid of the side effects	8	5	10	12	0.08	0.44 (0.17-1.13)	0.09	0.36 (0.14-0.96)	**0.04**
I'm afraid of needles	5	5	4	6	0.90	1.10 (0.38-3.20)	0.87	0.83 (0.29-2.42)	0.74
I prefer alternative (homeopathic) medicine to vaccines	7	3	10	13	**0.01**	0.28 (0.09-0.83)	**0.02**	0.21 (0.07-0.65)	**0.01**
I have never had the flu	13	11	17	6	**0.03**	0.60 (0.32-1.11)	0.10	1.91 (0.72-5.05)	0.19
The vaccine will make me sick with the flu	7	8	6	8	0.70	1.36 (0.61-3.05)	0.45	0.95 (0.40-2.28)	0.91

I got a flu shot the year before so I didn't need it	1	1	2	0	0.26	0.47 (0.15-1.51)	0.21	1.77 (0.21-14.84)	0.60
Another reason	26	25	21	39	**0.01**	1.29 (0.77-2.14)	0.33	0.53 (0.31-0.91)	**0.02**

Red text indicates survey items reflecting negative vaccine attitudes

[a] Column percentages (of vaccinated), weighted according to survey weights to achieve national representativeness

[b] Column percentages (of corresponding hesitancy categories) (except for first row "All" which is a row percentage), weighted according to survey weights to achieve national representativeness

[c] using the Pearson chi-square test at significance level of alpha=5%; bold indicates statistical significance ($p<0.05$)

[d] Construct scales combine scores for each relevant survey item (reversing negative items) and divide by maximum (e.g., 100 being complete trust and 0 being complete distrust); after dichotomizing at median, binary variable represents high vs low score (e.g., 1 being high trust and 0 being low trust)

[e] Likert scale response options (strongly agree, agree, disagree, strongly disagree, don't know) dichotomized to agree/disagree (don't know coded as disagree), results for agreement shown; other scale response options dichotomized to reflect affirmative/negative, results for affirmative shown

[i] asked only to vaccinated respondents

[j] Odds Ratio (95% Confidence Interval) of hesitating vs not hesitating before receiving COVID-19 vaccine for affirmative survey response vs not

[k] Reference category for logistic regression of categorical variables
