# Supplementary material for: COVID-19 Vaccination Status, Attitudes, and Values among US Adults in September 2021
Source: J Clin Med. 2022 Jun 28;11(13):3734. doi: 10.3390/jcm11133734 (PMC9267733; doi:10.3390/jcm11133734)
Supplement: Supplementary file 1 [file jcm-11-03734-s001.zip › Table S3.pdf]

Table S3. Frequency and Odds of COVID-19 Vaccination among Hesitant by Vaccine Attitudes, Trust in CDC and HDs, and Sociodemographic Characteristics

Numbers in the "Total" column indicate the percentage of the hesitant (either unvaccinated or vaccinated after hesitation) weighted sample providing the September 2021 survey response in each row. Numbers in the "Hesitation Prior to Vaccination" columns indicate the percentage of those whose amount of hesitation prior to receiving the COVID-19 vaccine match that of the column header who provided the survey response in each row. The numbers in the "OR (95%CI)" columns indicate the Odds Ratio comparing the different amounts of hesitation matching that of the column header by the survey response in each row. The numbers in the "P-value" columns indicate the p-value of the associations described in the columns to the left, boldface indicating statistical significance (p<0.05).

Survey Items	Total Hesitant (%)[a]	Hesitation Prior to Vaccination, %[b]			p-value[c]	Some Hesitation vs. Unvaccinated		A Lot of Hesitation vs. Unvaccinated	
		Some	A Lot	Still Unvaccinated		OR (95%CI)[j]	p-value[c]	OR (95%CI)[j]	p-value[c]
**All**	100	37	17	46					
**Constructs** [d]									
Confidence in vaccines	36	65	27	16	**<0.01**	9.47 (6.58-13.64)	**<0.01**	1.91 (1.21-3.03)	**0.01**
Trust in the Centers for Disease Control and Prevention (CDC)	24	42	23	11	**<0.01**	5.98 (4.06-8.80)	**<0.01**	2.54 (1.56-4.14)	**<0.01**
Trust in local and state health departments (HDs)	28	44	25	17	**<0.01**	3.69 (2.60-5.24)	**<0.01**	1.60 (1.02-2.49)	**0.04**

**Sociodemographic Characteristics**									
Gender					0.53				
Female	55	55	59	53		ref [k]		ref [k]	
Male	45	45	41	47		0.95 (0.70-1.29)	0.73	0.80 (0.54-1.18)	0.26
Age (years)					**<0.01**				
18-29	25	22	16	30		ref [k]		ref [k]	
30-44	30	27	31	32		1.18 (0.75-1.84)	0.48	1.86 (1.01-3.44)	**0.05**
45-59	25	26	30	22		1.59 (1.01-2.51)	0.05	2.63 (1.42-4.86)	**<0.01**
60+	20	26	23	15		2.36 (1.48-3.76)	**<0.01**	2.92 (1.55-5.52)	**<0.01**
Education (attained)					**<0.01**				
<High School	12	10	12	13		ref [k]		ref [k]	
High School	34	29	32	38		0.99 (0.58-1.67)	0.96	0.91 (0.48-1.74)	0.78
Some College	29	27	28	31		1.12 (0.66-1.89)	0.68	0.97 (0.51-1.84)	0.93
Bachelors or Higher	25	34	28	17		2.51 (1.45-4.36)	**<0.01**	1.79 (0.92-3.50)	0.09
Race/Ethnicity					**0.01**				
White, non-Hispanic	60	54	62	64		ref [k]		ref [k]	
Black, non-Hispanic	16	15	17	15		1.16 (0.81-1.66)	0.42	1.18 (0.74-1.88)	0.48

Hispanic	17	20	15	16		1.53 (1.08-2.18)	**0.02**	1.00 (0.63-1.57)	0.98
Other, non-Hispanic	7	11	6	5		2.84 (1.41-5.69)	**<0.01**	1.31 (0.49-3.50)	0.59
Region					0.07				
Northeast	17	17	24	14		ref [k]		ref [k]	
Midwest	21	18	24	22		0.66 (0.39-1.11)	0.11	0.64 (0.36-1.15)	0.14
South	39	39	34	42		0.76 (0.49-1.19)	0.23	0.49 (0.29-0.83)	**0.01**
West	23	25	18	22		0.94 (0.57-1.53)	0.80	0.50 (0.27-0.90)	**0.02**
Metropolitan Statistical Area status (metro vs non-metro)	84	88	85	81	0.10	1.66 (1.03-2.67)	**0.04**	1.29 (0.73-2.26)	0.38
Employment status (working vs not working)	66	65	65	66	0.97	0.97 (0.71-1.33)	0.84	0.96 (0.65-1.44)	0.86
Household income					**<0.01**				
<$50k	38	35	31	43		ref [k]		ref [k]	
$50-85k	33	31	39	31		1.24 (0.87-1.78)	0.23	1.76 (1.12-2.75)	**0.01**
$85-150k	17	15	19	18		1.05 (0.66-1.66)	0.85	1.49 (0.84-2.63)	0.17
$150k+	12	18	11	8		2.83 (1.72-4.65)	**<0.01**	1.90 (0.96-3.76)	0.07

Household size					**0.01**				
1	15	16	19	12		ref [k]		ref [k]	
2	31	33	39	27		0.89 (0.56-1.41)	0.61	0.91 (0.53-1.56)	0.72
3	19	18	15	22		0.61 (0.37-1.03)	0.06	0.43 (0.22-0.83)	**0.01**
4+	35	33	27	38		0.64 (0.41-1.02)	0.06	0.45 (0.25-0.79)	**0.01**
Number of children (ages 2-17)					0.09				
0						ref [k]		ref [k]	
1	67	68	100	1		0.76 (0.48-1.21)	0.25	0.73 (0.40-1.34)	0.31
2	13	11	76	100		0.82 (0.52-1.30)	0.40	0.46 (0.24-0.91)	**0.02**
3+	12	13	12	62		0.76 (0.44-1.30)	0.31	0.35 (0.15-0.78)	**0.01**
Political affiliation					**<0.01**				
Republican	32	27	35	34		ref [k]		ref [k]	
Democrat	25	32	22	19		2.09 (1.40-3.12)	**<0.01**	1.13 (0.67-1.88)	0.65
Independent	30	29	26	32		1.15 (0.77-1.73)	0.49	0.78 (0.48-1.28)	0.33
Something else	14	11	17	15		0.88 (0.53-1.46)	0.62	1.05 (0.59-1.87)	0.87
Physical health (good vs not good)	83	82	78	84	0.22	0.85 (0.57-1.26)	0.41	0.66 (0.41-1.06)	0.09

**Affirmative Responses to Survey Items** [e]									
COVID-19 Disease									
Have you ever had COVID-19?	24	17	22	30	**<0.01**	0.47 (0.31-0.70)	**<0.01**	0.64 (0.39-1.03)	0.06
How likely do you think it is that you will have COVID-19 over the next year?	22	18	22	24	0.19	0.70 (0.48-1.03)	0.07	0.87 (0.54-1.40)	0.57
When indoors in a crowded setting do you (or would you) wear a mask?	73	86	79	60	**<0.01**	4.17 (2.84-6.13)	**<0.01**	2.52 (1.59-4.00)	**<0.01**
I am concerned that I or my family/friends will be exposed when others do not wear masks in public.	46	61	43	35	**<0.01**	2.85 (2.06-3.95)	**<0.01**	1.35 (0.90-2.01)	0.14
COVID-19 Vaccine									
How important do you think a COVID-19 vaccine is to stop the spread of infection in the US?	65	96	76	37	**<0.01**	38.29 (20.31-72.19)	**<0.01**	5.37 (3.47-8.31)	**<0.01**
Are you worried that the COVID-19 vaccine is not safe for adults?	49	16	50	74	**<0.01**	0.07 (0.05-0.10)	**<0.01**	0.35 (0.23-0.52)	**<0.01**
Have you discussed getting vaccinated with your healthcare provider?	32	34	36	28	0.10	1.34 (0.96-1.85)	0.08	1.46 (0.97-2.19)	0.07
Of those who have: the provider	49	66	52	32	**<0.01**	4.11 (2.30-7.35)	**<0.01**	2.32 (1.17-4.58)	**0.02**

encouraged getting the vaccine.									
COVID-19 in Children									
COVID-19 can be a serious disease for some children.	77	89	83	65	**<0.01**	4.43 (2.89-6.80)	**<0.01**	2.64 (1.64-4.23)	**<0.01**
I am concerned about the safety of COVID-19 vaccine in children.	82	73	85	87	**<0.01**	0.41 (0.28-0.62)	**<0.01**	0.89 (0.52-1.52)	0.66
Vaccinating children against COVID-19 is important to end the pandemic and get back to normal.	48	79	52	22	**<0.01**	13.67 (9.45-19.79)	**<0.01**	3.88 (2.59-5.82)	**<0.01**
It is better for children to develop immunity to COVID-19 by getting sick rather than by getting a shot.	47	22	46	67	**<0.01**	0.14 (0.10-0.19)	**<0.01**	0.41 (0.28-0.61)	**<0.01**
COVID-19 in children is no worse than a cold or the flu.	47	30	47	62	**<0.01**	0.26 (0.19-0.36)	**<0.01**	0.56 (0.38-0.82)	**<0.01**
Vaccines Other than COVID-19									
Had flu vaccination, past 12 months.	35	54	38	18	**<0.01**	5.42 (3.82-7.69)	**<0.01**	2.85 (1.87-4.36)	**<0.01**
Of parents: Have you ever delayed having your child get a shot other than the flu for	21	12	9	30	**<0.01**	0.31 (0.15-0.63)	**<0.01**	0.24 (0.07-0.76)	**0.02**

reasons other than illness or allergy?									
Of parents: Have you ever decided not to have your child get a shot other than the flu for reasons other than illness or allergy?	16	9	12	21	**0.03**	0.38 (0.17-0.84)	**0.02**	0.51 (0.18-1.46)	0.21
Have you or anyone you know ever had a serious reaction to a vaccine?	15	5	12	24	**<0.01**	0.18 (0.11-0.30)	**<0.01**	0.43 (0.24-0.76)	**<0.01**
Healthcare and Science in General									
Received high quality care from healthcare provider, past 12 months.	87	91	82	86	**0.01**	1.80 (1.07-3.02)	**0.03**	0.75 (0.42-1.33)	0.32
In general, would you say that you trust science?	83	92	81	77	**<0.01**	3.42 (2.12-5.53)	**<0.01**	1.26 (0.79-2.02)	0.32
Political Activities and Support									
People may be involved in civic and political activities. In the past 12 months, have you...									
Attended a political protest or rally	5	45	11	44	0.38	1.32 (0.65-2.72)	0.44	0.66 (0.25-1.76)	0.41
Contacted a government official	12	34	17	49	0.78	0.85 (0.53-1.37)	0.51	0.88 (0.49-1.6)	0.68
Volunteered or worked for a Presidential	1	30	16	54	0.85	0.7 (0.21-2.36)	0.57	0.78 (0.14-4.22)	0.77

campaign									
Volunteered or worked for a political candidate other than a Presidential campaign	1	25	30	46	0.47	0.68 (0.17-2.79)	0.60	1.73 (0.40-7.41)	0.46
Volunteered or worked for a political party, issue, or cause	2	35	17	49	0.98	0.9 (0.33-2.5)	0.84	0.9 (0.25-3.29)	0.88
Served on a committee for a civic, non-profit or community organization	3	40	14	46	0.85	1.11 (0.51-2.4)	0.80	0.82 (0.31-2.2)	0.70
Written a letter or email to a newspaper/magazine or called a live radio or TV show	4	30	18	51	0.72	0.73 (0.34-1.59)	0.43	0.93 (0.37-2.31)	0.87
Commented about politics on a message board or internet site	17	34	17	49	0.68	0.84 (0.56-1.26)	0.39	0.93 (0.57-1.51)	0.77
Shared your opinion about a town or community issue at a public meeting	4	39	16	45	0.93	1.11 (0.50-2.46)	0.81	0.91 (0.31-2.69)	0.86
Held a publicly elected office	0	28	0	72	0.63	0.49 (0.04-6.24)	0.58		
Signed a petition	24	38	18	44	0.80	1.12 (0.79-1.59)	0.52	1.09 (0.71-1.69)	0.68
Ran for a publicly elected office	0	100	0	0	0.46				
None of these	64	37	17	46	0.98	1 (0.73-1.37)	0.99	0.96 (0.65-1.42)	0.84
Do you identify with or actively support									

any of the following political movements?										
Tea Party (Taxed Enough Already)	9	31	19	51	0.56	0.74 (0.41-1.32)	0.30	0.96 (0.49-1.9)	0.92
Environmental Rights	13	48	14	38	**0.02**	1.69 (1.08-2.65)	**0.02**	0.94 (0.52-1.69)	0.84
Women's Rights/ Me Too	17	45	15	40	0.07	1.52 (1.02-2.27)	0.04	1 (0.58-1.71)	1.00
Racial Equality	21	48	14	38	**<0.01**	1.78 (1.23-2.56)	**<0.01**	0.99 (0.61-1.61)	0.96
Right to Life	19	30	15	54	0.05	0.64 (0.43-0.94)	**0.02**	0.7 (0.42-1.15)	0.15
Peace/Anti-War	10	51	13	36	**0.02**	1.88 (1.15-3.06)	**0.01**	0.95 (0.46-1.98)	0.90
Lesbian, Gay, Bisexual, Transgender, Queer (LGBTQ) Rights	13	47	17	36	**0.04**	1.8 (1.11-2.91)	**0.02**	1.3 (0.71-2.39)	0.40
Indivisible	2	44	1	55	0.18	1.01 (0.31-3.27)	0.99	0.07 (0.01-0.58)	**0.01**
Black Lives Matter	18	45	15	40	**0.04**	1.55 (1.06-2.26)	**0.02**	0.96 (0.58-1.58)	0.88
Men's Rights	5	36	15	49	0.88	0.91 (0.45-1.86)	0.80	0.8 (0.33-1.91)	0.61
Alt-right	1	0	16	84	0.11			0.52 (0.08-3.42)	0.49
Boogaloo movement	0	0	47	53	0.32			2.31 (0.20-26.22)	0.50
Antifa	1	84	13	2	**<0.01**	43.44 (5.37-351.23)	**<0.01**	14.32 (1.28-160.03)	**0.03**
QAnon	2	15	13	71	0.17	0.27 (0.06-1.28)	0.10	0.50 (0.10-2.45)	0.39
Anti-gun violence	10	53	16	31	**<0.01**	2.33 (1.45-3.74)	**<0.01**	1.42 (0.74-2.7)	0.29
None of these	56	35	19	45	0.22	0.94 (0.69-1.28)	0.69	1.33 (0.90-1.96)	0.15
Do you identify with or actively support any of the following organizations?									

National Rifle Association (NRA)	18	28	17	55	**0.03**	0.57 (0.38-0.86)	**0.01**	0.81 (0.49-1.33)	0.41
Heritage Foundation	4	43	24	33	0.28	1.63 (0.75-3.55)	0.22	1.99 (0.77-5.16)	0.15
Planned Parenthood	12	44	15	41	0.29	1.40 (0.89-2.21)	0.14	1.00 (0.53-1.87)	0.99
National Right to Life Committee	7	35	15	50	0.81	0.86 (0.47-1.57)	0.62	0.81 (0.39-1.71)	0.58
Greenpeace	4	35	20	45	0.90	0.99 (0.49-2.02)	0.99	1.21 (0.47-3.12)	0.70
Sierra Club	4	59	10	32	**0.02**	2.43 (1.11-5.32)	**0.03**	0.83 (0.26-2.67)	0.76
Amnesty International	3	63	9	28	**0.02**	2.96 (1.15-7.66)	**0.03**	0.88 (0.22-3.52)	0.86
National Education Association									
Foundation	6	35	12	53	0.54	0.82 (0.41-1.63)	0.57	0.57 (0.19-1.69)	0.31
American Civil Liberties Union (ACLU)	5	54	15	31	**0.03**	2.31 (1.16-4.63)	**0.02**	1.33 (0.56-3.14)	0.51
Americans for Prosperity	2	21	17	62	0.32	0.42 (0.13-1.34)	0.14	0.74 (0.20-2.71)	0.64
MoveOn.org	3	52	13	35	0.22	1.89 (0.80-4.45)	0.14	1.01 (0.34-3.05)	0.98
The NAACP/National Association for									
the Advancement of Colored People	9	42	16	41	0.46	1.32 (0.83-2.09)	0.23	1.05 (0.57-1.94)	0.86
American Red Cross	20	42	15	43	0.33	1.27 (0.87-1.85)	0.21	0.93 (0.56-1.54)	0.76
Chamber of Commerce	3	38	17	45	0.98	1.07 (0.45-2.56)	0.87	0.99 (0.31-3.19)	0.99
Freedom Caucus	2	42	12	47	0.77	1.13 (0.46-2.73)	0.79	0.68 (0.17-2.68)	0.58
None of these	56	35	18	46	0.44	0.90 (0.67-1.22)	0.50	1.17 (0.79-1.73)	0.43

Sources of Health Information

Which of the following sources have you used to look for health and wellness related information or education in the past 12 months?									
Doctor	51	43	19	38	**<0.01**	2.10 (1.54-2.85)	**<0.01**	1.77 (1.20-2.6)	**<0.01**
Pharmacist	18	43	21	37	**0.04**	1.58 (1.06-2.36)	**0.03**	1.62 (0.99-2.65)	0.06
Nurse, nurse practitioner or physician's assistant	25	41	20	40	0.12	1.38 (0.96-1.99)	0.08	1.45 (0.93-2.25)	0.10
Relative, friend or co-worker	24	36	18	46	0.98	0.98 (0.69-1.41)	0.93	1.03 (0.66-1.62)	0.89
Someone you know who has a particular medical condition	10	36	17	47	0.97	0.94 (0.56-1.59)	0.82	0.95 (0.50-1.81)	0.88
Disease-related association or society	5	56	13	32	**0.03**	2.29 (1.09-4.81)	**0.03**	1.03 (0.41-2.62)	0.94
Patient support group or foundation	2	46	9	45	0.52	1.29 (0.46-3.64)	0.63	0.5 (0.10-.10-2.57)	0.41
Educational forum at a local clinic, hospital, community center or other location	4	30	24	46	0.50	0.79 (0.35-1.79)	0.58	1.38 (0.58-3.29)	0.46
Pharmaceutical company	1	33	21	46	0.91	0.89 (0.25-3.15)	0.86	1.22 (0.30-5.01)	0.78
Health insurance company	6	55	15	30	**0.01**	2.42 (1.26-4.66)	**0.01**	1.36 (0.62-2.96)	0.45
Newspapers or magazines	5	44	22	34	0.26	1.66 (0.80-3.41)	0.17	1.79 (0.77-4.15)	0.17
Television	6	34	16	50	0.83	0.86 (0.47-1.57)	0.61	0.85 (0.41-1.77)	0.66

The internet	45	39	17	44	0.41	1.23 (0.90-1.66)	0.19	1.07 (0.73-1.57)	0.73
Social Media (such as Facebook, Twitter)	7	38	17	45	0.97	1.07 (0.58-1.98)	0.83	1.02 (0.49-2.12)	0.95
Healthcare app for smartphone or tablet	6	30	26	44	0.22	0.82 (0.42-1.62)	0.57	1.59 (0.79-3.21)	0.20
Have not looked for information in the past 12 months	27	28	16	57	**<0.01**	0.51 (0.36-0.72)	**<0.01**	0.63 (0.40-0.98)	**0.04**
Barriers, Specific Concerns and Other Reasons For Not Getting the Flu Vaccine									
Of those who did not get a flu shot this past year: this is because… [i]									
The flu is not a serious illness	11	19	16	65	0.50	0.64 (0.29-1.4)	0.26	0.90 (0.41-1.96)	0.79
I'm healthy	25	24	14	61	0.77	0.89 (0.54-1.46)	0.64	0.82 (0.45-1.48)	0.51
I just didn't think about it	20	36	9	56	**0.01**	1.66 (1.03-2.68)	**0.04**	0.52 (0.24-1.11)	0.09
I didn't know where to get it	1	13	0	87	0.37	0.34 (0.05-2.47)	0.29		
I didn't have health insurance	3	38	2	60	0.07	1.45 (0.59-3.54)	0.42	0.12 (0.02-0.96)	**0.05**
I didn't have time	4	67	6	27	**<0.01**	6.25 (2.33-16.73)	**<0.01**	0.8 (0.16-4.05)	0.79
I don't believe in vaccines	8	3	10	87	**<0.01**	0.08 (0.03-0.22)	**<0.01**	0.37 (0.15-0.92)	**0.03**
I'm afraid of the side effects	13	19	15	66	0.36	0.64 (0.34-1.23)	0.18	0.79 (0.39-1.59)	0.51
I'm afraid of needles	4	26	21	53	0.73	1.15 (0.42-3.15)	0.79	1.51 (0.55-4.12)	0.42
I prefer alternative (homeopathic)	13	19	15	66	0.31	0.61 (0.32-1.17)	0.13	0.81 (0.40-1.67)	0.57

medicine to vaccines									
I have never had the flu	13	33	7	59	0.05	1.36 (0.79-2.34)	0.26	0.43 (0.17-1.07)	0.07
The vaccine will make me sick with the flu	10	15	13	72	0.06	0.44 (0.21-0.90)	**0.03**	0.63 (0.28-1.39)	0.25
I got a flu shot the year before so I didn't need it	3	15	2	83	**0.01**	0.39 (0.14-1.13)	0.08	0.11 (0.01-0.84)	**0.03**
Another reason	28	19	22	58	**0.01**	0.69 (0.43-1.11)	0.12	1.68 (1.01-2.78)	**0.04**

Red text indicates survey items reflecting negative vaccine attitudes

[a] Column percentages (of hesitant), weighted according to survey weights to achieve national representativeness

[b] Column percentages (of corresponding hesitancy categories) (except for first row "All" which is a row percentage), weighted according to survey weights to achieve national representativeness

[c] using the Pearson chi-square test at significance level of alpha=5%; bold indicates statistical significance ($p<0.05$)

[d] Construct scales combine scores for each relevant survey item (reversing negative items) and divide by maximum (e.g., 100 being complete trust and 0 being complete distrust); after dichotomizing at median, binary variable represents high vs low score (e.g., 1 being high trust and 0 being low trust)

[e] Likert scale response options (strongly agree, agree, disagree, strongly disagree, don't know) dichotomized to agree/disagree (don't know coded as disagree), results for agreement shown; other scale response options dichotomized to reflect affirmative/negative, results for affirmative shown

[j] Odds Ratio (95% Confidence Interval) of hesitating before vaccinating vs not yet vaccinating for affirmative survey response vs not

[k] Reference category for logistic regression of categorical variables
