# Supplementary material for: COVID-19 Vaccination Status, Attitudes, and Values among US Adults in September 2021
Source: J Clin Med. 2022 Jun 28;11(13):3734. doi: 10.3390/jcm11133734 (PMC9267733; doi:10.3390/jcm11133734)
Supplement: Supplementary file 1 [file jcm-11-03734-s001.zip › Table S6.pdf]

Table S6. Age by Vaccine Attitudes, Trust in CDC and HDs, and Sociodemographic Characteristics

Numbers in the "Total" column indicate the percentage of the total weighted sample providing the September 2021 survey response in each row. Numbers in the "Age" columns indicate the percentage of those whose age match that of the column header who provided the survey response in each row. The numbers in the final column indicate the p-value of this association, boldface indicating statistical significance (p<0.05).

Survey Items	Total (%)[a]	Age (years) (%)[b] 18-29	30-44	45-59	60+	p-value[c]
**All**	100	20	25	24	30	
**Constructs** [d]						
Confidence in vaccines	65	58	59	64	75	**<0.01**
Trust in the Centers for Disease Control and Prevention (CDC)	47	40	42	47	55	**<0.01**
Trust in local and state health departments (HDs)	46	39	39	47	55	**<0.01**
**Sociodemographic Characteristics**						
Gender						0.58
Female	52	49	50	52	54	
Male	48	51	50	48	46	
Education (attained)						**<0.01**
<High School	9	10	12	10	7	
High School	28	31	20	28	32	
Some College	28	27	26	27	30	
Bachelors or Higher	35	33	42	35	30	
Race/Ethnicity						**<0.01**
White, non-Hispanic	63	53	56	64	74	
Black, non-Hispanic	12	14	13	12	10	

Hispanic	17	22	21	7	6	
Other, non-Hispanic	8	11	10	17	9	
Region						0.29
Northeast	17	15	16	21	17	
Midwest	21	18	22	20	22	
South	38	43	35	36	38	
West	24	24	26	23	23	
Metropolitan Statistical Area status (metro vs non-metro)	87	86	88	89	84	0.24
Employment status (working vs not working)	62	70	77	79	31	**<0.01**
Household income						**0.01**
<$50k	30	34	29	27	32	
$50-85k	31	34	29	30	33	
$85-150k	19	18	21	18	18	
$150k+	20	14	21	26	17	
Household size						**<0.01**
1	17	14	11	17	23	
2	35	25	23	31	57	
3	19	21	23	20	14	
4+	29	40	44	33	6	
Number of children (ages 2-17)						**<0.01**
0	73	81	41	69	96	
1	11	8	19	15	2	
2	11	7	24	11	1	

3+	6	4	17	4	0	
Political affiliation						**<0.01**
Republican	26	18	20	31	32	
Democrat	34	36	36	32	33	
Independent	30	34	31	26	29	
Something else	10	12	13	11	6	
Physical health (good vs not good)	84	88	83	84	83	0.24
**Affirmative Responses to Survey Items** [e]						
COVID-19 Disease						
Have you ever had COVID-19?	18	21	23	19	12	**<0.01**
How likely do you think it is that you will have COVID-19 over the next year?	20	21	29	19	12	**<0.01**
When indoors in a crowded setting do you (or would you) wear a mask?	80	77	80	78	84	0.06
I am concerned that I or my family/friends will be exposed when others do not wear masks in public.	61	61	60	59	63	0.56
COVID-19 Vaccine						
Have you received a COVID-19 vaccine?	77	66	71	79	88	**<0.01**
How important do you think a COVID-19 vaccine is to stop the spread of infection in the US?	82	77	80	82	88	**<0.01**
Are you worried that the COVID-19 vaccine is not safe for adults?	26	32	31	27	17	**<0.01**
Have you discussed getting vaccinated with your healthcare provider?	36	28	29	34	50	**<0.01**

Of those who have: the provider encouraged getting the vaccine.	70	59	62	67	80	**<0.01**
COVID-19 in Children						
COVID-19 can be a serious disease for some children.	86	84	86	85	87	0.56
I am concerned about the safety of COVID-19 vaccine in children.	62	64	67	60	57	**0.01**
Vaccinating children against COVID-19 is important to end the pandemic and get back to normal.	71	67	66	71	76	**0.01**
It is better for children to develop immunity to COVID-19 by getting sick rather than by getting a shot.	29	32	32	30	22	**<0.01**
COVID-19 in children is no worse than a cold or the flu.	32	36	32	33	27	0.05
Vaccines Other than COVID-19						
Had flu vaccination, past 12 months.	55	41	48	54	71	**<0.01**
Of parents: Have you ever delayed having your child get a shot other than the flu for reasons other than illness or allergy?	16	19	16	16	2	0.35
Of parents: Have you ever decided not to have your child get a shot other than the flu for reasons other than illness or allergy?	11	19	9	11	7	0.16
Have you or anyone you know ever had a serious reaction to a vaccine?	8	9	10	9	7	0.26
Healthcare and Science in General						
Received high quality care from healthcare provider, past 12 months.	91	90	90	89	95	**0.01**
In general, would you say that you trust science?	90	88	92	90	90	0.33
Among Vaccinated: Boosters[j]						

If the CDC were to recommend a booster dose so your body can continue to protect you against COVID-19, how likely are you to get one? [j]	87	84	86	83	91	**0.02**
Of Unvaccinated: Mandates and Incentives [i]						
Of employed: If my employer required me to get the COVID-19 vaccine... I would get vaccinated. [i]	14	17	14	10	5	0.29
Of those who would not:						
I would quit my job. [i]	29	41	24	19	34	**0.02**
I would protest. [i]	32	28	34	34	34	0.84
I would consider legal action. [i]	43	28	46	52	62	**0.01**
I am not sure what I would do. [i]	42	47	47	32	31	0.16
If I was offered a $25-$100 gift card for getting fully vaccinated... I would be more likely to get vaccinated. [i]	5	5	8	3	2	0.21
If I was automatically enrolled in a lottery when I got fully vaccinated that made me eligible to win at least $100K... I would be more likely to get vaccinated. [i]	6	6	6	7	2	0.54
Seeing fewer people wear masks in public makes me more likely to get vaccinated. [i]	13	18	11	11	8	0.19
Of Unvaccinated: Knowledge and Decision-Making re: COVID-19 Vaccination [i]						
I am knowledgeable about COVID-19 vaccines for adults. [i]	75	74	68	83	82	0.05
I still have many unanswered questions about COVID-19 vaccines for adults. [i]	68	70	65	72	68	0.69
I still cannot decide whether getting the COVID-19 vaccine is best for me. [i]	47	47	49	45	50	0.91
Talking with other people is important in helping me make up	35	41	32	34	34	0.51

my mind about COVID-19 vaccination for myself. [i]						
Of Unvaccinated: Specific Concerns and Other Reasons For Not Getting a COVID-19 Vaccine [i]						
How fast COVID-19 vaccines were developed and made available to the public. [i]	59	55	50	70	68	**0.01**
COVID-19 vaccines are new. [i]	80	79	76	85	81	0.46
The safety of COVID-19 vaccines has not been studied for a long enough period of time. [i]	83	72	82	92	92	**<0.01**
A lot of people who get the vaccine feel tired, achy and get headaches and fever. [i]	74	74	72	74	75	0.94
Some people have bad allergic reactions to COVID-19 vaccines. [i]	77	74	76	77	87	0.20
I am not sure the ingredients in COVID-19 vaccines are safe. [i]	76	71	71	79	90	**0.01**
COVID-19 vaccines might change my genes or DNA (cause mutations). [i]	33	29	29	42	34	0.16
COVID-19 vaccines might affect my fertility or ability to have children. [i]	34	39	38	27	24	0.08
There were not enough people of my race/ethnicity who were a part of the vaccine studies. [i]	16	15	13	21	17	0.38
They are experimenting on people with the COVID-19 vaccine. [i]	69	64	67	72	77	0.30
The drug companies are making a lot of money off of COVID-19 vaccines. [i]	70	63	64	79	86	**<0.01**
Some COVID-19 vaccines are made from aborted fetuses. [i]	23	22	20	26	28	0.61
I have a health condition that might make me at increased risk of having a bad reaction to the COVID-19 vaccine. [i]	29	22	25	39	32	0.05
I have a health condition that would prevent the COVID-19	16	14	14	22	16	0.47

vaccine from being effective. [i]						
I am worried about severe vaccine side effects such as myocarditis (heart swelling), Guillain Barre Syndrome (paralysis), or severe blood clots. [i]	74	71	73	78	79	0.52
Vaccine recommendations are influenced more by politics than by science. [i]	83	80	78	88	91	**0.04**
I am worried about the safety of COVID-19 vaccines. [i]	86	84	84	88	89	0.73
I do not trust how quickly the COVID-19 vaccine was developed. [i]	86	85	87	88	85	0.88
I worry that I would have a reaction to the vaccine. [i]	78	82	79	77	69	0.31
I worry about having to provide personal information (name, address, phone number, insurance card) to get the vaccine. [i]	26	26	24	28	28	0.86
Those I trust (friends, family, or religious leaders) do not want to get the vaccine. [i]	53	56	54	54	46	0.62
I have seen posts on social media that make me wary of the vaccine. [i]	51	63	49	47	38	**0.01**
I want to wait to see what happens to others who are vaccinated. [i]	73	80	71	74	62	0.08
I need more time to learn and think more about it. [i]	59	67	60	58	45	0.05
Of pregnant women: I do not think the COVID-19 vaccine is safe for me or my baby. [i]	39	48	36	0	39	0.24
Of Unvaccinated: Reasons to Get COVID-19 Vaccination [i]						
COVID-19 vaccines are likely to protect me from the COVID-19 strains circulating. [i]	24	29	24	27	11	**0.03**
COVID-19 vaccines are likely to protect me from new variants of COVID-19 that may appear in the future. [i]	22	31	21	17	13	**0.02**
It's important for me to get vaccinated so I don't accidentally	21	34	19	19	6	**<0.01**

give COVID-19 to other people in my family. [i]						
It's important for me to get vaccinated to help get my community back to normal. [i]	17	27	16	10	8	**<0.01**
The government is acting in my or my family's best interest when it comes to COVID-19. [i]	18	21	19	16	16	0.75
Of Unvaccinated: Barriers to COVID-19 Vaccination [i]						
I cannot get transportation to where COVID-19 vaccines are being given. [i]	5	9	4	2	1	**0.02**
The times when COVID-19 vaccines are being given conflict with my daily schedule. [i]	12	20	9	8	4	**<0.01**
I do not know how to register to get a vaccine appointment. [i]	11	21	7	10	5	**<0.01**
I know how to register to get a vaccine appointment but it is too difficult. [i]	14	22	12	10	4	**<0.01**
I cannot miss work to get vaccinated. [i]	18	30	15	15	6	**<0.01**
At least one of the above. [i]	34	50	31	29	14	**<0.01**
Political Activities and Support						
People may be involved in civic and political activities. In the past 12 months, have you...						
Attended a political protest or rally	7	10	6	6	4	**0.01**
Contacted a government official	15	13	16	14	18	0.18
Volunteered or worked for a Presidential campaign	3	4	2	2	3	0.15
Volunteered or worked for a political candidate other than a Presidential campaign	2	4	1	2	3	**0.03**
Volunteered or worked for a political party, issue, or cause	4	5	3	3	4	0.29
Served on a committee for a civic, non-profit or community organization	5	4	3	6	7	**0.04**

Written a letter or email to a newspaper/magazine or called a live radio or TV show	4	5	4	3	5	0.68
Commented about politics on a message board or internet site	19	23	21	16	18	0.09
Shared your opinion about a town or community issue at a public meeting	4	4	4	4	5	0.78
Held a publicly elected office	0	0	0	0	1	0.39
Signed a petition	26	32	24	22	26	**0.03**
Ran for a publicly elected office	0	0	0	0	0	0.63
None of these	60	58	63	63	57	0.11
Do you identify with or actively support any of the following political movements?						
Tea Party (Taxed Enough Already)	6	7	4	5	8	0.12
Environmental Rights	20	28	22	14	18	**<0.01**
Women's Rights/ Me Too	23	35	28	16	18	**<0.01**
Racial Equality	26	35	29	20	23	**<0.01**
Right to Life	15	14	11	14	18	**0.03**
Peace/Anti-War	11	15	15	8	9	**<0.01**
Lesbian, Gay, Bisexual, Transgender, Queer (LGBTQ) Rights	19	33	23	14	12	**<0.01**
Indivisible	2	2	2	1	2	0.75
Black Lives Matter	25	40	29	17	18	**<0.01**
Men's Rights	4	5	5	3	4	0.42
Alt-right	1	1	1	0	1	0.84
Boogaloo movement	0	0	1	0	0	0.05
Antifa	2	4	4	2	1	**<0.01**
QAnon	1	1	1	1	1	0.77
Anti-gun violence	16	20	16	11	18	**<0.01**
None of these	53	44	52	58	55	**<0.01**
Do you identify with or actively support any of the following						

organizations?						
National Rifle Association (NRA)	13	9	11	15	16	**0.02**
Heritage Foundation	3	2	2	4	5	**0.03**
Planned Parenthood	19	29	22	12	14	**<0.01**
National Right to Life Committee	5	4	3	5	8	**0.02**
Greenpeace	6	6	6	5	7	0.59
Sierra Club	8	9	7	7	8	0.80
Amnesty International	6	6	6	6	6	0.97
National Education Association Foundation	6	9	5	6	6	0.16
American Civil Liberties Union (ACLU)	12	13	14	10	11	0.30
Americans for Prosperity	1	2	1	1	1	0.40
MoveOn.org	7	7	6	5	8	0.30
The NAACP/National Association for the Advancement of Colored People	12	20	11	9	10	**<0.01**
American Red Cross	23	25	21	21	25	0.31
Chamber of Commerce	3	2	3	4	4	0.35
Freedom Caucus	2	2	2	3	2	0.69
None of these	53	50	56	56	50	0.12
Sources of Health Information						
Which of the following sources have you used to look for health and wellness related information or education in the past 12 months?						
Doctor	60	55	57	58	68	**<0.01**
Pharmacist	21	18	20	20	26	**0.04**
Nurse, nurse practitioner or physician's assistant	29	29	24	29	32	0.11
Relative, friend or co-worker	23	34	26	21	16	**<0.01**
Someone you know who has a particular medical condition	8	12	9	7	7	**0.04**

Disease-related association or society	5	7	5	5	5	0.51
Patient support group or foundation	2	2	2	2	2	0.89
Educational forum at a local clinic, hospital, community center or other location	3	6	3	4	2	**0.01**
Pharmaceutical company	1	1	1	1	2	0.15
Health insurance company	7	7	6	6	9	0.16
Newspapers or magazines	7	7	6	5	8	0.17
Television	6	6	6	6	8	0.60
The internet	48	50	50	52	43	**0.03**
Social Media (such as Facebook, Twitter)	6	10	6	5	3	**<0.01**
Healthcare app for smartphone or tablet	6	7	5	5	6	0.62
Have not looked for information in the past 12 months	21	21	23	20	19	0.49
Barriers, Specific Concerns and Other Reasons For Not Getting the Flu Vaccine						
Of those who did not get a flu shot this past year: this is because… [i]						
The flu is not a serious illness	9	9	13	8	7	0.20
I'm healthy	22	26	24	21	17	0.22
I just didn't think about it	22	32	23	18	14	**<0.01**
I didn't know where to get it	1	2	1	1	0	0.23
I didn't have health insurance	3	3	5	3	1	0.15
I didn't have time	5	8	6	4	2	0.07
I don't believe in vaccines	6	7	4	7	7	0.39
I'm afraid of the side effects	11	13	10	8	14	0.30
I'm afraid of needles	4	7	5	3	2	0.19
I prefer alternative (homeopathic) medicine to vaccines	11	11	10	11	11	0.97
I have never had the flu	13	11	12	11	19	0.11

The vaccine will make me sick with the flu	9	7	8	12	11	0.32
I got a flu shot the year before so I didn't need it	2	3	2	3	1	0.34
Another reason	27	24	29	29	27	0.62

Red text indicates survey items reflecting negative vaccine attitudes

[a] Column percentages (of total sample), weighted according to survey weights to achieve national representativeness

[b] Column percentages (of age categories) (except for first row "All" which is a row percentage), weighted according to survey weights to achieve national representativeness

[c] using the Pearson chi-square test at significance level of alpha=5%; bold indicates statistical significance ($p<0.05$)

[d] Construct scales combine scores for each relevant survey item (reversing negative items) and divide by maximum (e.g., 100 being complete trust and 0 being complete distrust); after dichotomizing at median, binary variable represents high vs low score (e.g., 1 being high trust and 0 being low trust)

[e] Likert scale response options (strongly agree, agree, disagree, strongly disagree, don't know) dichotomized to agree/disagree (don't know coded as disagree), results for agreement shown; other scale response options dichotomized to reflect affirmative/negative, results for affirmative shown

[i] asked only to unvaccinated respondents

[j] asked only to vaccinated respondents
